# Transcriptome and metabolome profiling unveil the accumulation of chlorogenic acid in autooctoploid Gongju

**DOI:** 10.3389/fpls.2024.1461357

**Published:** 2024-11-01

**Authors:** Li Zhao, Yu Cao, Gaomeng Shan, Jiayi Zhou, Xintong Li, Peng Liu, Yansong Wang, Songhao An, Ri Gao

**Affiliations:** ^1^ College of Agricultural, Yanbian University, Yanji, Jilin, China; ^2^ Yanbian Academy of Forestry Sciences, Yanji, Jilin, China; ^3^ Department of Chemistry, Yanbian University, Yanji, Jilin, China

**Keywords:** Gongju, transcriptome, metabolomic, chlorogenic acid, transcription factor

## Abstract

**Background:**

Gongju is recognized as one of the four traditional Chinese medicinal herbs, and its main constituents are chlorogenic acid (CGA) and its derivative material. CGA content in autooctoploid Gongju are considerably elevated compared with those in parental tetraploid Gongju at different flowering stages. However, the underlying molecular mechanisms governing the regulation CGA content remain poorly understood.

**Methods:**

Therefore, we conducted integrated transcriptome and metabolome analyses of different flowering stages in autooctoploid and tetraploid Gongju to elucidate the underlying molecular mechanisms governing CGA biosynthesis.

**Results:**

Transcriptome analysis showed that the number of differentially expressed genes in the budding stage (BS), early flowering stage (EF), and full flowering stage (FF) of tetraploid and octoploid Gongju were 3859, 11,211, and 6837, respectively. A total of 563, 466, and 394 differential accumulated metabolites were respectively identified between the bud stages of tetraploid and octoploid Gongju (4BS vs. 8BS), between the early flowering stages of tetraploid and octoploid Gongju (4EF vs. 8EF), and the full flowering stages of tetraploid and octoploid Gongju (4FF vs. 8FF) groups. The integrated analysis of transcriptomics and metabolomics revealed that the expression of pma6460 and mws0178, which are key enzymes involved in the CGA synthesis pathway, increased during the flowering stages of octoploid Gongju relative to that of tetraploid Gongju. The expression levels of *CmHQT* and *CmC3H* genes associated with CGA synthesis were higher in octoploid plants than in tetraploid plants at various flowering stages. To investigate the potential regulation of transcription factors involved in CGA synthesis, we analyzed the coexpression of *CmC3H* and *CmHQT* with *CmMYBs* and *CmbHLHs*. Results indicated that transcription factors, such as *CmMYB12* (Cluster-30519.0), *CmMYB26* (Cluster-75874.0), *CmMYB5* (Cluster-94106.0), *CmMYB1* (Cluster-71968.7), *CmbHLH62* (Cluster-32024.1), *CmbHLH75* (Cluster-62341.0), *CmbHLH62* (Cluster-32024.8), *CmbHLH75* (Cluster-60210.0), and *CmbHLH16* (Cluster-90665.1) play a pivotal role in CGA synthesis regulation. The present study provides novel insights into the molecular mechanisms underlying CGA accumulation in autopolyploid Gongju.

## 1 Introduction

Gongju is a perennial herb belonging to the Chrysanthemum family, exhibiting large flowers with wide petals and refreshing fragrance. Its dried capitulum contains phenolic acid, flavonoids, organic acids, volatile oils, amino acids, and other chemical components, possessing multifunctional properties encompassing not only ornamental value but also applications in tea preparation and medicinal practices. The chrysanthemum tea can be categorized into fetal chrysanthemum (budding stages) and flowering chrysanthemum (early flowering and full flowering stages), with varying levels of active substances at different stages of flowering. The quality of Gongju is primarily determined by the levels of chlorogenic acid (CGA), 3,5-O-dicafeoyl quinic acid (ICGA), and luteolin. However, Gongju cultivation mainly involves long-term propagation through branching, which reduces product quality and stress resistance. That is, CGA content is reduced.

**Figure 1:**
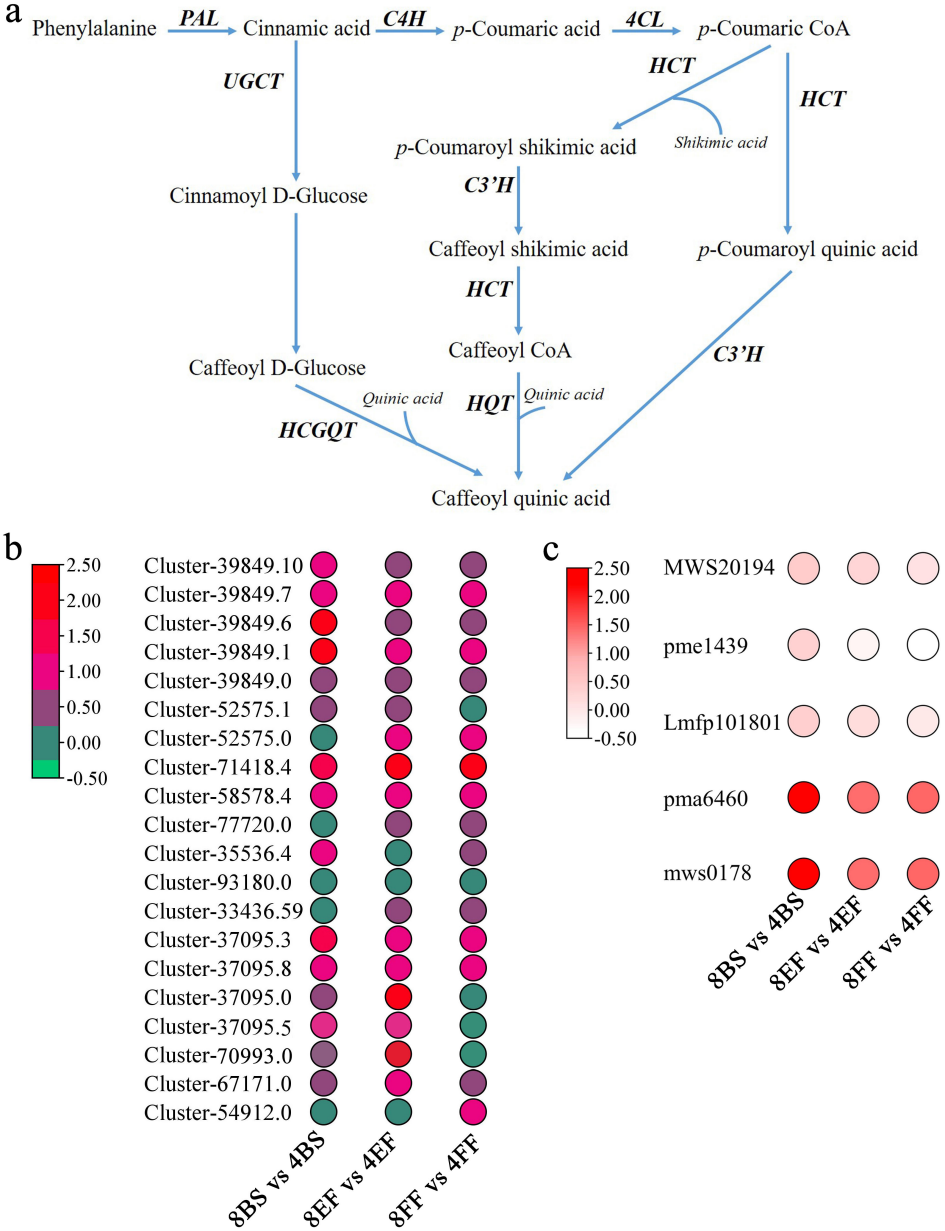
Profiles of DEGs and DAMs in CGA biosynthesis pathways of tetraploid and octoploid Gongju during flowering. **(A)** Chlorogenic acid anabolic pathway **(B)** DEGs: APL: Cluster-39849.10, Cluster-39849.7, Cluster-39849.6, Cluster-39849.1; Cluster-39849.0; C4H: Cluster-52575.0, Cluster-52575.1; 4CL: Cluster-71418.4, Cluster-58578.4, Cluster-77720.0, Cluster-35536.4, Cluster-93180.0, Cluster-33436.59; HQT: Cluster-37095.3, Cluster-37095.8, Cluster-37095.0, Cluster-37095.5, Cluster-70993.0; C3H: Cluster-67171.0, Cluster-54912.0; **(C)** DAMs: MWS20194: Cinnamic acid; pme1439: p-Coumaric acid; Lmfp101801: Caffeoylshikimic acid; pma6460: 4-O-p-Coumaroylquinic acid; mws0178: Chlorogenic acid.

CGA is a vital secondary metabolite widely distributed in various vegetables and fruit. Chrysanthemum, coffee, and honeysuckle exhibit high CGA content (Hu et al., 2009; Xia et al., 2019), and various crops, such as tomatoes, potatoes, peaches, and apples contain CGA (Chen et al., 2020b). Notably, CGA exhibits diverse biological activities, such as scavenging of free radicals and modulating blood pressure and lipid levels, and antibacterial and anti-inflammatory properties (Santana-Gálvez et al., 2017). Thus, CGA synthesis has been a focus of research. CGA biosynthesis proceeds through the phenylpropane metabolic pathway. Phenylalanine is initially oxidized to cinnamic acid by the enzyme phenylalanine ammonia-lyase (PAL), and cinnamic acid then undergoes three synthesis pathways (Su et al., 2022). Enzymes involved in CGA synthesis include cinnamate 4-hydroxylase (C4H), 4-hydroxycinnamoyl-CoA ligase (4CL), hydroxycinnamoyl-CoA shikimate/quinate hydroycinnamoyltransferase (HCT/HQT), and 4-coumarate3-hydroxylase (C3H) enzymes. HQT and C3H are considered rate-limiting enzymes (**Figure 1A**) (Payyavula et al., 2014). The expression of *C3H* and *HQT* exerts considerable effects on the biosynthesis of GCA in rice, apple, and other plant species (Zhang et al., 2018; Su et al., 2023; Wang et al., 2023).

The metabolic pathway of phenylalanine is regulated by multiple genes. Previous studies have demonstrated that the expression levels of *MYB*, *bHLH*, and *WD* transcription factors exert regulatory control over the synthesis of *p*-coumaric CoA and flavone (Yue et al., 2023). Notably, *MYB* and *bHLH* transcription factors play pivotal roles in the regulation of CGA synthesis. In carrot, potato, and *Lonicera macranthoides*, *MYB1*, *MYB3*, *MYB5*, and *MYB15* act as transcriptional activators by binding to the promoters of *PAL* and *4CL* genes, modulating the phenolic acid pathway and promoting the biosynthesis of CGA (Tuan et al., 2014; Bartley et al., 2016; Tang et al., 2021). The transcription factor *TabHLH1* directly binds to the motifs of *Ta4CLpro*, resulting in a substantial increase in CGA content in the overexpressed *TabHLH1* transgenes of *Taraxacum antungense* (Liu et al., 2021a). However, the molecular mechanisms regulating CGA synthesis in chrysanthemum remain poorly understood.

Polyploidy is prevalent in angiosperms and has been crucial for the formation, diversification, and evolution of plant species. According to chromosomal origin, polyploids can be classified into allopolyploids, which consist of chromosomes from different species, and autopolyploids, which comprise chromosomes from the same species. The induction of autopolyploidy is primarily achieved through the application of mutagens, such as colchicine, which effectively inhibits spindle filament formation and facilitates the process. Compared with their parents, autopolyploid plants exhibit an increase in secondary metabolite content and undergo morphological changes, demonstrating enhanced adaptability to extreme environments. An autopolyploid possesses a diverse range of characteristics that mainly affect dosage after genome doubling and epigenetic modification and ultimately lead to the differential expression of functional genes. The autopolyploid of *Panicum virgatum* L., *Isatidis Radix*, rose, and *Cannabis sativa* demonstrated the elevated synthesis of secondary metabolites and improved salinity tolerance in comparison with their respective parental species. These differences are attributable to the differential expression patterns of genes associated with these traits (Chen et al., 2020a; Zhang et al., 2021b; Tang et al., 2023). In our previous study, the successful induction of Gongju autooctoploid was achieved through colchicine treatment. Remarkably, the autoctoploid capitulum exhibited enhanced enlargement and increased CGA content compared with its parental plant. However, the literature available on the enhancement of CGA content in autooctoploid plant is limited. Therefore, in this study, we employed a combination of transcriptome and metabolome analyses to investigate the differentially expressed genes (DEGs) and differential accumulated metabolites (DAMs) between octoploid and tetraploid Gongju at various stages of flowering, and identified the key genes associated with CGA synthesis during different flowering stages of octoploid Gongju. Imultaneously, we screen for co-expressed transcription factors alongside *CmHQT* and *CmC3H* as pivotal genes.

## 2 Materials and methods

### 2.1 Plant materials

Gongju autooctoploid and its parental seedlings were propagated through cutting and subsequently cultivated in a greenhouse at Yanbian University Teaching Base in Yanji City, Jilin Province, China. The environmental conditions were as follows: temperature of 25–27°C, photoperiod of 14/10 h (light/dark), and relative humidity of 60%–70%. The uppermost flowers were collected and snap-frozen in liquid nitrogen during the budding, early flowering, and full flowering stages. One portion of each flower was utilized for transcriptome analysis, and another portion was subjected to desiccation for the analysis of secondary metabolites. Each treatment was performed with three biological replicates.

### 2.2 Quantification of CGA and ICGA content

The flowers of Gongju autooctoploid and its parent were collected at the budding stage (BS), early flowering (EF), and full flowering stages (FF). Subsequently, they were ground into powder and subjected to low-temperature drying. Each powder sample (0.25 g) was accurately weighed and transferred to a conical flask, and then 25 mL of chromatographic grade methanol solution (70%) was added. Ultrasonic treatment was performed for 40 min (120 w, 40 kHz). Afterward, the sample was subjected to filtration using a microporous filter membrane prior to determination. Standards for CGA and ICAA were provided by Solaibao Technology Co., LTD. CGA and ICGA were quantified using high-performance liquid chromatography (Agilent 1269) following the established chromatographic conditions as described in Yu et al. (2021).

### 2.3 Transcriptome analysis

Total RNA was extracted from the frozen samples with a Trans-Zol plant kit (TIANGEN, Beijing) according to the manufacturer’s instructions. RNA quality was assessed through agarose gel electrophoresis. mRNA was enriched from total RNA with oligo (dT). A Clontech SMARTer PCR cDNA synthesis kit (TaKaRa, Dalian, China) was used for first-strand cDNA synthesis. Library construction and transcriptome sequencing were commercially provided by Metware Co., Ltd. (www.metware.cn). cDNA libraries underwent high-throughput sequencing performed on the Illumina Hiseq 4000 platform. The filtering process was performed using Cutadap, and high-quality reads were obtained. The obtained clean reads were aligned to the reference genome (http://210.22.121.250:8880/asteraceae/homePage) with HISAT2. Gene expression levels were evaluated using fragments per kilobase million (FPKM) values. Genes exhibiting a p-value <0.05 and |log2FC| > 1 were pairwise compared for the identification of DEGs by DESeq2. GO and KEGG enrichment analyses were performed on the annotated DEGs using topGo and clusterprofiler packages, respectively. The transcriptome datasets have been deposited in the NCBI database under the accession number PRJNA1154322.

### 2.4 Metabolome profiling

To investigate variations in metabolites in the budding, early flowering, and full flowering stages of Gongju autooctoploidy, we performed metabolic analyses on the three biological replicates of each sample at each stage. The samples were dispatched to Metware Biotechnology Ltd. (Wuhan, China) for the comprehensive analysis of secondary metabolites. The freeze-dried flowers were pulverized using a mixer mill at 30 Hz for 2 minutes. Subsequently, 100 mg of the resulting powder was weighed and subjected to overnight extraction at 4°C with 10 mL of methanol (70% concentration). Then the sample extracts were analyzed using an LC-ESI-Q TRAP-MS/MS system. The differential metabolites were identified by maximizing the dissimilarities between the metabolites of two samples through orthogonal projections to latent structures-discriminant analysis (OPLS-DA). The variable importance in projection (VIP) obtained from the OPLS-DA model was employed for multivariate analysis and initial screening of differential metabolites. We selected metabolites with a VIP ≥0.8 and | Log _2_ (fold change) | ≥0.48 as the differential metabolites for subsequent analysis.

### 2.5 Integrated transcriptomic and metabolomics analysis

The DEGs and DAMs were identified through comparisons between autooctoploid and tetraploid Gongju at various flowering stages. Each group had three biological replicates. Pearson correlation coefficients (PCCs) were calculated for the integration of the transcriptome and metabolome data at thresholds of >0.8 (PCC) and <0.05 (p-value). KEGG enrichment analysis was conducted according to the corresponding data DEGs and DAMs. Subsequently, a detailed investigation was performed on the metabolic pathway of CGA synthesis.

### 2.6 Weighed gene coexpression network analysis

To further investigate the crucial *CmHQT* (Cluster- 57865.0) and *CmC3H* (Cluster- 54912.0) genes involved in CGA synthesis, which are associated with the transcription factors *CmMYBs* and *CmbHLHs*, we constructed a weighted gene coexpression network with the R software WGCNA package. *CmHQT* or *CmC3H* were imported into WGCNA with *CmMYBs* and *CmbHLHs*, respectively, for the construction of coexpression modules. The expression correlation coefficients of the remaining genes were subsequently computed for the identification of an appropriate soft threshold for constructing gene networks with a scale-free topology model. Then, the modules containing *CmHQT* or *CmH3C* were identified, and their weight coefficients with *CmbHLHs* or *CmMYBs* were evaluated. The coexpression network was visualized using Cytoscape v3.8.0 at a threshold of 0.25.

### 2.7 Quantitative real-time PCR analysis

To validate the accuracy of transcriptome data, we conducted quantitative real-time PCR (qRT-PCR analysis) to assess the relative expression levels of diverse genes. A 20 μL reaction mixture comprising 10 μL of 2× SuperReal PreMix Plus (TIANGENG, China), each primer at a concentration of 10 µM, 0.4 μL of 50× ROX reference dye, 1 μL of cDNA template, and ddH_2_O was prepared. The specific primers were designed using Primer 5.0 software (**Supplementary Table S1**). Elongation factor I alpha (*EF1α*) and photosynthesis-related plastid gene representing photosystem I (*PSAA*) were considered the two reference genes for normalization (Wang et al., 2015). The relative expression levels of genes were analyzed using the 2^−ΔΔct^ method. All data analyses were performed with three biological replicates. Statistical analysis for significant differences was conducted using Excel 2019 and SPSS Statistics 22.0 software.

## 3 Results

### 3.1 Quantification of CGA and ICGA in tetraploid and octoploid Gongju at different stages of flowering

**Figure 2:**
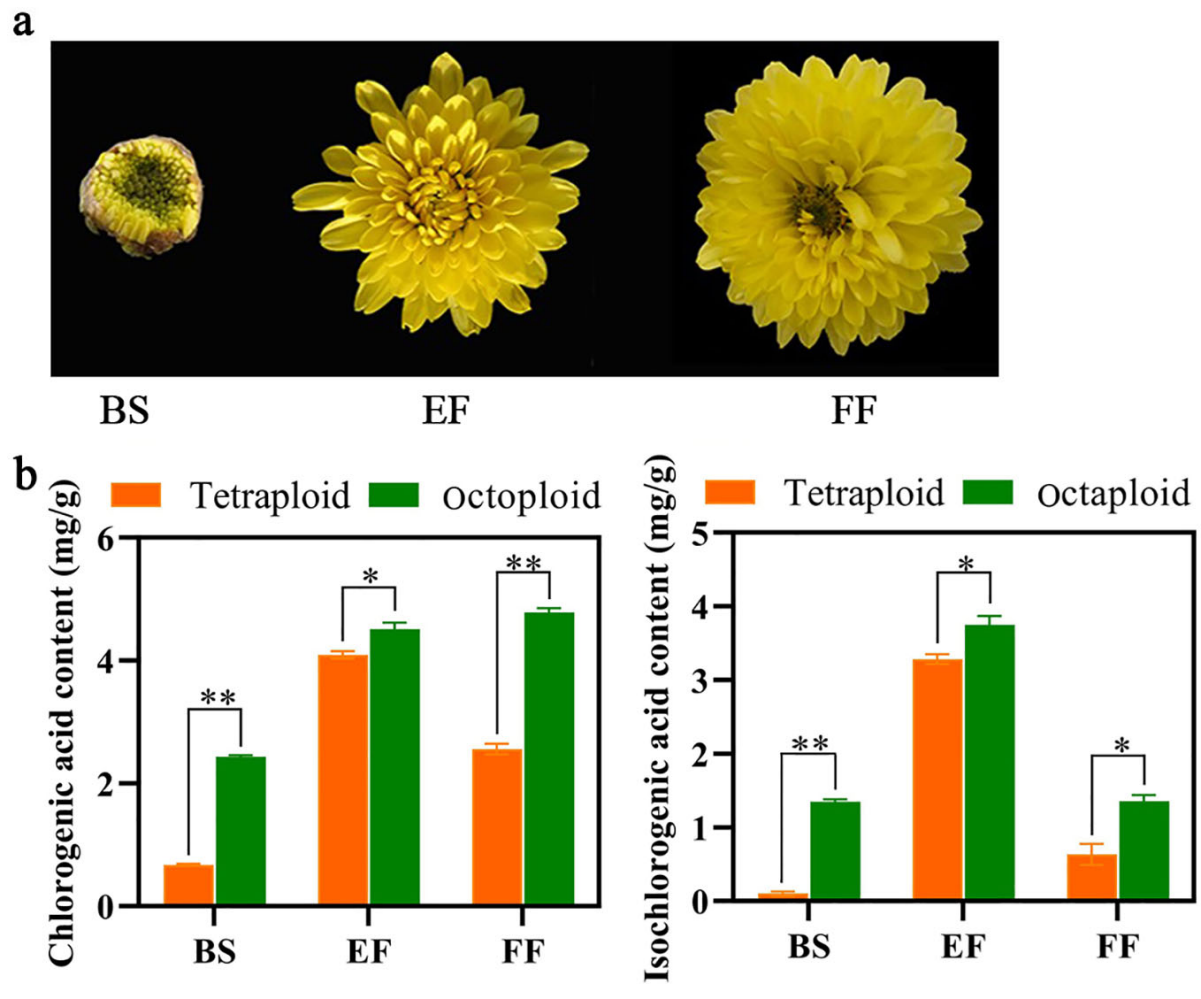
Quantification of CGA and ICGA in tetraploid and octoploid Gongju at various stages of the flowering process. **(A)** Different flowering periods of Gong octaploid. Budding stage (BS), early flowering stage (EF) and full flowering stage (FF). **(B)** Content of CGA and ICGA in tetraploid and octoploid Gongju. * shows a significant difference found at p≤ 0.05 and ** shows a significant difference found at p≤ 0.01 on the same flowering stage tested.

In tetraploid Gongju, the CGA and ICGA content exhibited an initial increase followed by a subsequent decrease, reaching peak values during the early flowering stage (4.3 mg/g and 3.8 mg/g, respectively). However, the CGA content demonstrated considerable increase at the early and FF stages, and ICGA levels were particularly elevated during the EF stage in octoploid Gongju. The levels of CGA and ICGA in octoploid Gongju were considerably higher than those observed during the corresponding flowering stage in tetraploid Gongju (**Figure 2**).

### 3.2 Identification, GO annotation, and GO enrichment of DEGs

**Figure 3:**
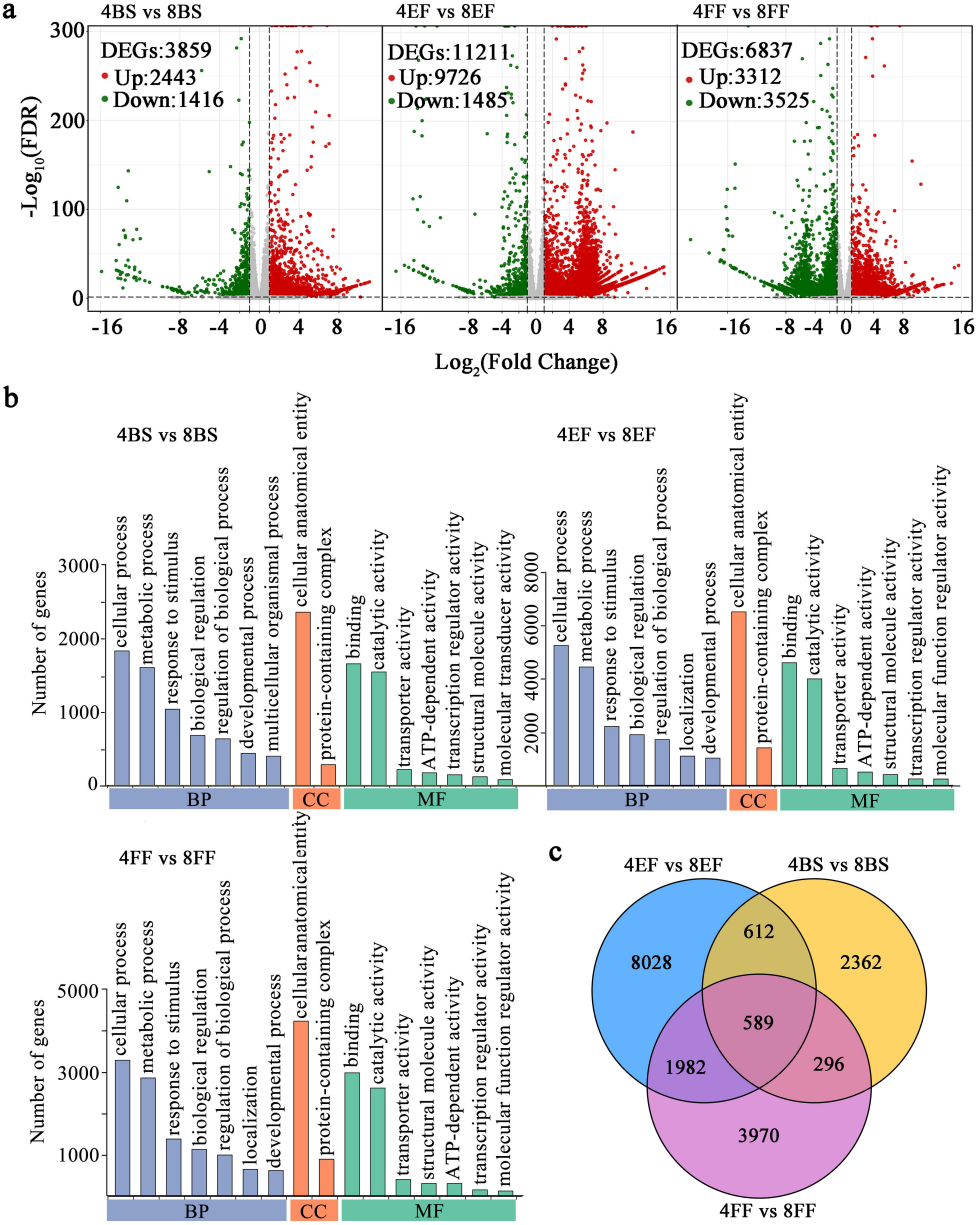
DEGs and GO enrichment in different flowering stage of tetraploid and octoploid Gongju. **(A)** Gene expression levels in volcano plots and the number of DEGs in the three comparison groups. The color red indicates upregulation, while green signifies downregulation, and gray represents no significant change. **(B)** GO functional classification of DEGs in the comparisons of 4BS vs 8BS, 4EF vs 8EF, 4FF vs 8FF. Only the top 7 terms in each core category are listed. **(C)** Venn diagram overlapping and differentially enriched GO terms obtained from the comparisons of 4BS vs 8BS, 4EF vs 8EF, 4FF vs 8FF.

Eighteen RNA-seq libraries (4BS-1, 4BS-2, 4BS-3, 4EF-1, 4EF-2, 4EF-3, 4FF-1, 4FF -2, 4FF-3, 8BS-1, 8BS-2, 8BS-3, 8EF-1, 8EF-2, 8EF-3, 8FF-1, 8FF-2, and 8FF-3) were constructed and sequenced in tetraploid and octoploid Gongju at various flowering stages. Overview and validation of RNA-seq findings were performed, as presented in **Supplementary Table S2** and **Supplementary Figure S1**. The volcano plots revealed the identification of 3859 DEGs in the comparison between 4BS and 8BS, 2443 genes were upregulated, and 1416 genes were downregulated. The expression levels of 9726 genes were upregulated in the 8EF group, whereas the expression levels of 1485 genes were downregulated relative to those of the 4EF group. Meanwhile, we identified 6837 DEGs between the 4FF and 8FF groups, 3312 genes showed upregulated expression, and 3525 genes exhibited downregulated expression (**Figure 3A**). Gene ontology (GO) analysis was performed according to the DEGs identified through comparisons at the flowering stages of tetraploid and octoploid Gongju. In the comparison between 4FFB and 8FFB, between 4EF and 8EF, and between 4FF and 8FF, a total of 32, 39, and 32 GO terms were assigned to three fundamental categories: cellular component (CC), biological process (BP), and molecular function (MF). The number of DEGs involved in “transcription regulator activity,” “metabolic process,” and “regulation of biological process” were 2.81, 2.18; 2.76, 1.78; 2.71, 1.58 times higher in the comparisons between 4EF and 8EF, as well as between 4FF and 8FF, compared to those observed in the comparisons between 4BS and 8BS (**Figure 3B** and **Supplementary Table S3**). Two enriched terms in MF, including “unsaturated fatty acid biosynthetic process”, “unsaturated fatty acid metabolic process”, were shared across all comparisons. The “flavonoid metabolic process” enriched terms consisted of 38 genes, among which 30 exhibited upregulation in the 4FF vs. 8FF group (**Supplementary Table S4**). The Venn diagram revealed that 1201, 2571, and 885 DEGs were identified in the comparisons between 4BS and 8BS, between 4EF and 8EF, and between 4FF and 8FF. Additionally, a total of 589 DEGs were found to be common across all groups (**Figure 3C**).

### 3.3 KEGG pathway analysis of DEG

**Figure 4:**
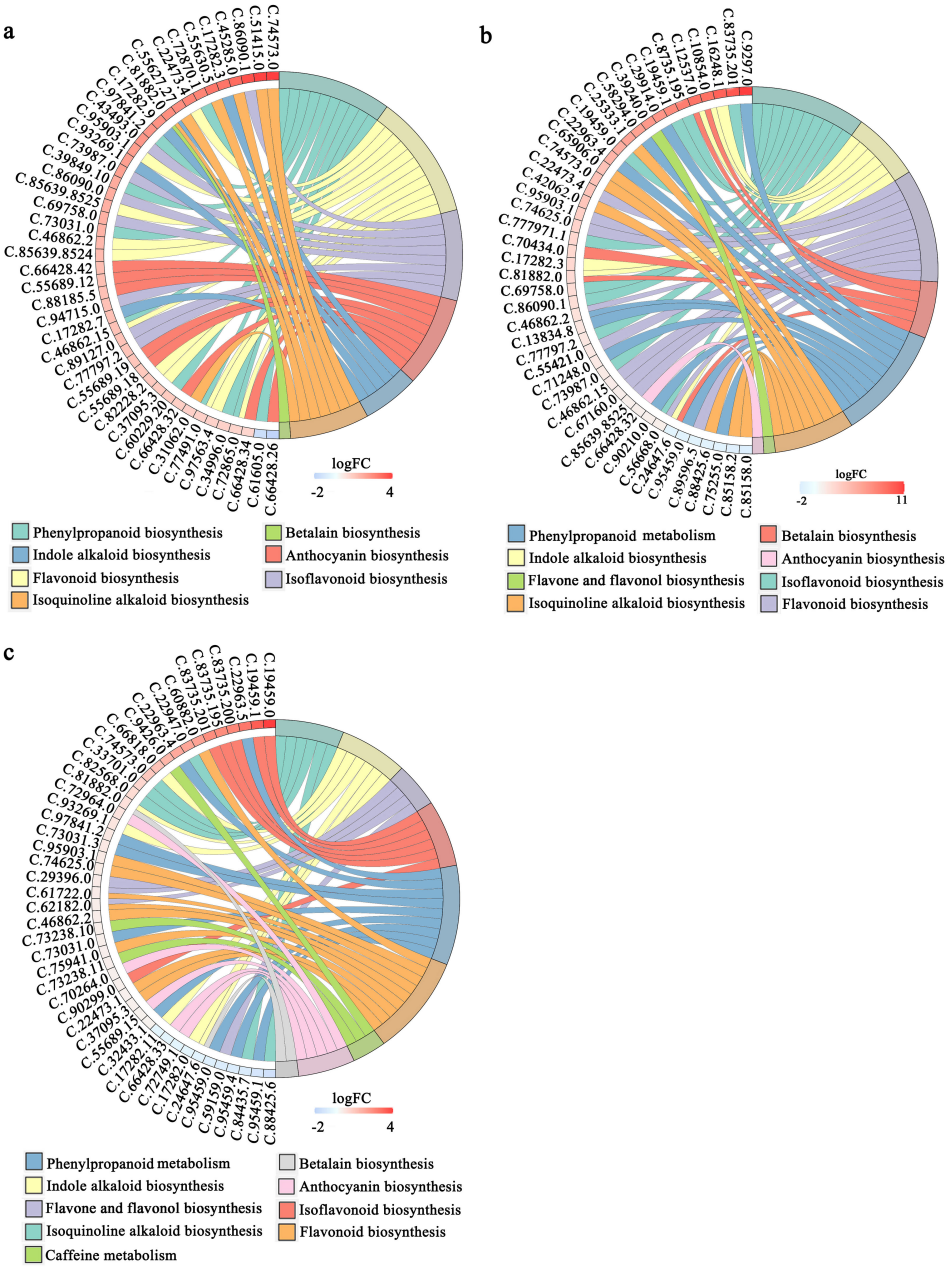
KEGG enrichment analysis of DEGs in different flowering stage of tetraploid and octoploid Gongju. **(A)** 4BS vs 8BS, **(B)** 4EF vs 8EF, **(C)** 4FF vs 8FF. SB, Bud stage; EF, Early flowering stage; FF, Full flowering stage; 4 and 8, tetraploid and octoploid. The metabolic pathways exhibiting a higher number of differentially expressed genes were enumerated.

Based on the KEGG enrichment analysis, we identified 2210, 4439, and 4146 DEGs in the comparisons between 4BS and 8BS, between 4EF and 8EF, and between 4FF and 8FF, respectively. The DEGs exhibited enrichment in 27, 40, and 43 KEGG pathways, respectively (**Supplementary Table S5**). In the enriched pathways, these combinations included phenylpropanoid biosynthesis, flavonoid biosynthesis, flavone and flavonol biosynthesis, and isoflavonoid biosynthesis pathways. In the enriched pathways, these combinations included phenylpropanoid biosynthesis, flavonoid biosynthesis, flavone and flavonol biosynthesis, and isoflavonoid biosynthesis pathways. The CGA enzyme catalyzes the synthesis of precursors involved in the phenylalanine metabolic pathway. Within the phenylpropanoid synthesis pathway, a total of 69, 31, and 29 differentially expressed genes were identified in the comparison between 4BS and 8BS groups, as well as between 4EF and 8EF groups, and finally between 4FF and 8FF groups (**Figure 4**; **Supplementary Table S5**).

### 3.4 Metabolomic analysis of Gongju at different ploidy levels and flowering stages

**Figure 5:**
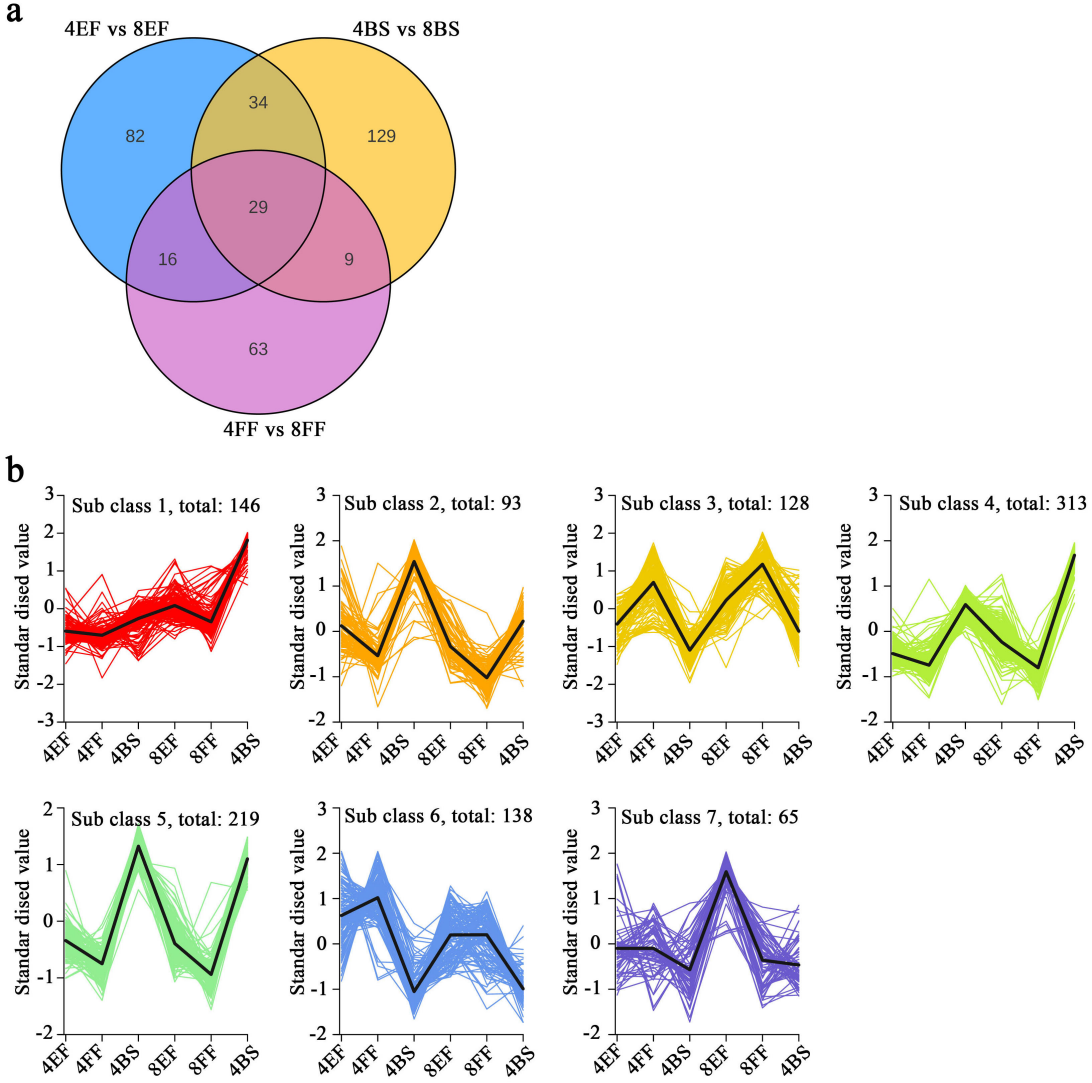
Metabolomic analyses of various flowering stages in Gongju. **(A)** Venn diagram analysis, which comprised three comparison groups: 4BS vs 8BS, 4EF vs 8EF, and 4FF vs 8FF **(B)** The K means analysis of DAMs.

To compare the disparity in secondary metabolites between tetraploid and octoploid Gongju at different stages of flowering, we conducted an analysis on metabolites. A preliminary understanding of the overall metabolome was obtained by conducting PCA on the dataset. The first principal component (PC1) accounted for 56.66% of the total variation, whereas the second principal component (PC2) explained 13.8% (**Supplementary Figure S2**). The samples were classified into six distinct groups, and the within-group repeatability was excellent. The obtained data held potential for further analysis. The analysis of DAMs revealed 563, 466, and 394 DAMs in the comparisons between 4BS and 8BS, between 4EF and 8EF, and between 4FF and 8FF, respectively (**Supplementary Table S6**). Venn diagram analysis revealed 63 DAMs, which were exclusively identified in the comparisons between 4BS and 8BS and between 4EF and 8EF. Moreover, 38 DAMs were exclusively found in the comparisons between 4BS and 8BS and between 4FF and 8FF. Lastly, a shared set of 29 DAMs was observed across all groups (**Figure 5A**). To gain further insights into the alterations in metabolic substance composition across different flowering stages, we standardized the DAM data and performed k-means clustering analysis. The DAMs exhibited seven distinct patterns of variation across the different flowering stages of Gongju and were categorized into subclasses 1–7. CGA and isochlorogenic acid and were categorized into subclasses 1 and 2. Cafeoyl quinic acid derivatives were categorized into subclasses 1, 3, 4, 5, and 7 (**Figure 5B**).

### 3.5 Combined analysis of DEMs and DEGs

**Figure 6:**
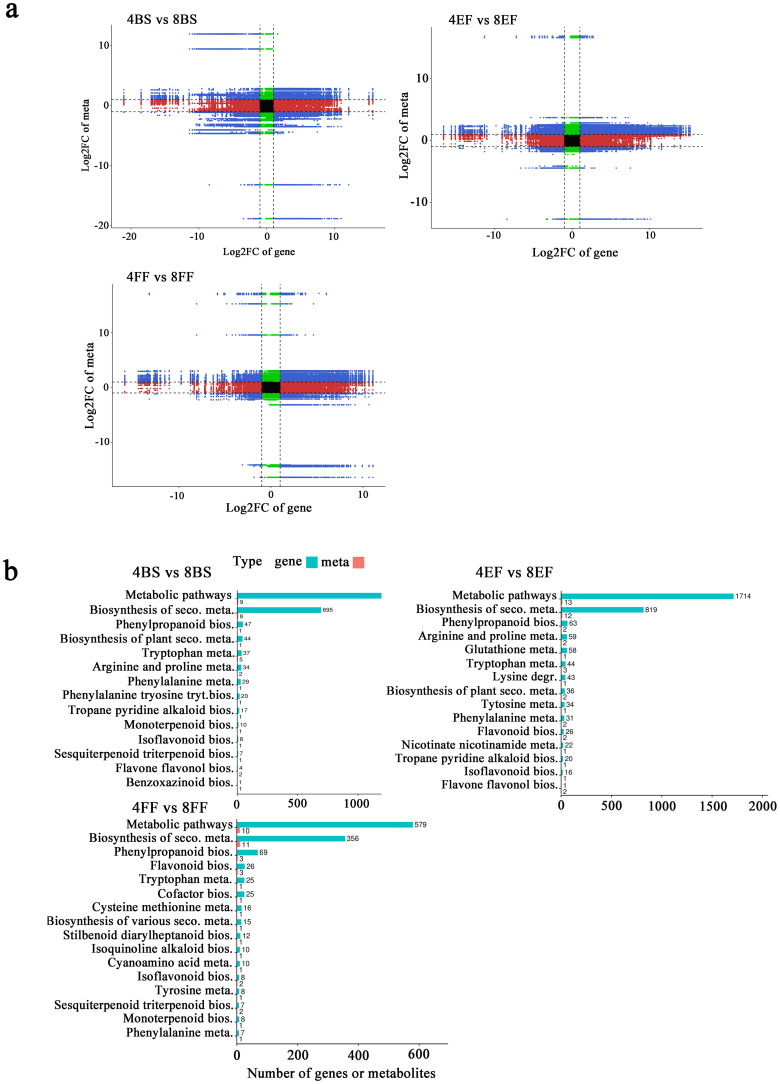
Transcriptomic and metabolic integration analysis of tetraploid and octoploid Gongju during flowering. **(A)** Nine-quadrant diagrams. three comparison groups: 4BS vs 8BS, 4EF vs 8EF, and 4FF vs 8FF. **(B)** KEGG pathway enrichment diagram.

The biosynthesis of bioactive compounds was comprehensively investigated between tetraploid and octoploid Gongju, resulting in the identification of 6837 DEGs and 117 DAMs in the comparison between 4BS and 8BS; 11,211 DEGs and 161 DAMs in the comparison between 4EF and 8EF; and 3859 DEGs and 201 DAMs in the comparison between 4FF and 8FF through integrated analysis (**Supplementary Table S7**). The nine-quadrant diagrams visually illustrate corresponding variations between DEGs and DAMs, suggesting the potential direct and indirect regulatory role of these DEGs in the alterations of corresponding metabolites (**Figure 6A**). Association analysis base on the KEGG database revealed considerable enrichment in interconnected DAMs and DEGs in 14 metabolic pathways in the comparison between 4BS and 8BS. In the comparisons between 4EF and 8EF and between 4FF and 8FF, enrichment was observed in 20 and 18 metabolic pathways (**Supplementary Table S8**). Additionally, the comparison between 4BS and 8BS revealed enriched pathways, including phenylpropanoid biosynthesis, tryptophan metabolism, arginine metabolism, phenylalanine metabolism, and phenylalanine, tyrosine, and tryptophan biosynthesis. The abundance of DEGs and DAMs involved in phenylpropanoid biosynthesis, arginine and proline metabolism, glutathione metabolism, tryptophan metabolism, and lysine degradation metabolic pathway were predominantly observed in the comparison between 4EF and 8EF. Phenylpropanoid biosynthesis, flavonoid biosynthesis, typtophan metabolism, cofactor biosynthesis, and cysteine methionine metabolism were enriched in the comparison between 4FF and 8FF. These findings suggested that these metabolic pathways serve as a means to enhance the availability of substances in octoploid Gongju (**Figure 6B**).

### 3.6 Profiles of DEGs and DAMs in CGA biosynthetic pathways

CGA is an important and effective component of Gongju and is mainly synthesized by the phenylalanine metabolic pathway. The contents of CGA (mws0178) and *p*-coumaroyl quinic acid (pma6460) in octoploid Gongju were 2.20, 1.4, 1.35, and 1.60, 1.35, 1.45 times higher than those in tetraploid Gongju at the budding, early flowering, and full flowering stages, respectively. Additionally, no obvious difference in *p*-coumaric acid (pme1439), cinnamic acid (MWS20194), and caffeoylshikimic acid (Lmfp101801) content was found between tetraploid and octoploid Gongju (**Figure 1C**). The corresponding genes involved in phenylalanine metabolism underwent considerable changes simultaneously. The expression levels of *APL* (Cluster-39849.10, Cluster-39849.7, and Cluster-39849.6), *C4H* (Cluster-52575.0), *4CL* (Cluster-71418.4 and Cluster-58578.4), *HQT* (Cluster-37095.3 and Cluster-37095.8), and *C3H* (Cluster-67171.0) genes in octoploid plants were considerably higher than those observed in tetraploid during the corresponding flowering stage (**Figure 1B**). In addition, the expression levels of Cluster-37095.8 and Cluster-67171.0 in octoploid Gongju at the first flowering stage were 2.3 and 1.8 times higher, respectively, compared to those in tetraploid Gongju. The implication of this finding is that these genes play a pivotal role in the biosynthesis of CGAs in octoploid Gongju.

### 3.7 Analysis of gene correlation network

**Figure 7:**
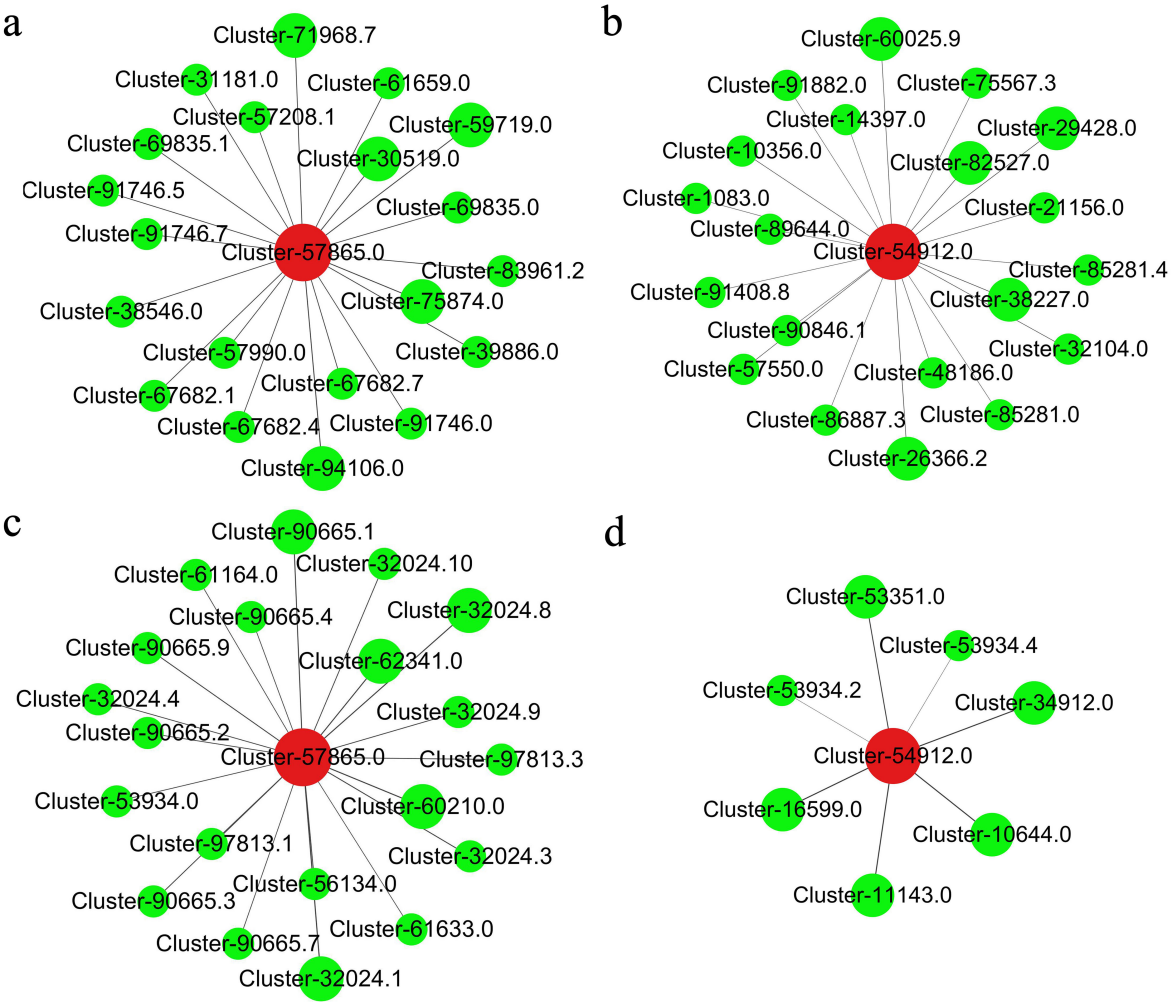
The co-expression analysis of transcription factors (*CmbHLHs* and *CmMYBs*) and genes *CmC3H* and *CmHQT* **(A, B)**: *CmC3H* and *CmHQT* with *CmMYBs*, respectively. **(C, D)**: *CmC3H* and *CmHQT* with *CmbHLHs*, respectively. Cluster- 57865.0: *CmHQT*; Cluster- 54912.0: *CmH3C*. Cluster-94106.0 (*CmMYB5*), Cluster-75874.0 (*CmMYB26*), Cluster-59719.0 (*CmMYB16*), Cluster-30519.0 (*CmMYB12*), and Cluster-71968.7 (*CmMYB1*); Cluster-26366.2 (*CmMYB3R*); Cluster-32024.1 (*CmbHLH62*), Cluster-60210.0 (*CmbHLH75*), Cluster-32024.8 (*CmbHLH62*), Cluster-62341.0 (*CmbHLH75*), and Cluster-90665.1 (*CmbHLH16*). The diameter of the circle was related to the weight coefficient, and the larger the diameter, the larger the weight coefficient.

The genes *CmC3H* and *CmHQT* play crucial roles in the synthesis of CGA. To elucidate the regulation of *CmC3H* and *CmHQT* expression by upstream transcription factors, a correlation network analysis was performed on *CmC3H* and *CmHQT* along with 404 putative regulatory *CmMYBs* and 126 candidate *CmbHLHs* involved in CGA biosynthes. A hierarchical cluster tree was constructed for the establishment of the relationships of *CmC3H* and *CmHQT* with *CmMYBs* and those of *CmC3H* and *CmHQT* with *CmbHLHs*. We obtained four modules for the relationships of *CmC3H* and *CmHQT* with *CmMYBs* and those of *CmC3H* and *CmHQT* with *CmbHLHs* (**Supplementary Figure S3**). *CmC3H* with 72 *CmMYBs* and *CmHQT* with 43 *CmMYBs* exhibited positive correlations with the blue and brown modules, respectively. Moreover, *CmC3H* and 7 *CmbHLHs* and *CmHQT* with 32 *CmbHLHs* demonstrated positive correlations with the brown and turquoise modules, respectively. The top five correlations of *CmC3H* and *CmHQT* with *CmbHLHs* were Cluster-10644.0, Cluster-11143.0, Cluster-16599.0, Cluster-34912.0, and Cluster-53351.0 and Cluster-32024.1 (*CmbHLH62*), Cluster-60210.0 (*CmbHLH75*), Cluster-32024.8 (*CmbHLH62*), Cluster-62341.0 (*CmbHLH75*), and Cluster-90665.1 (*CmbHLH16*). The top five correlations of *CmHQT* with *CmMYBs* were identified as Cluster-94106.0 (*CmMYB5*), Cluster-75874.0 (*CmMYB26*), Cluster-59719.0 (*CmMYB16*), Cluster-30519.0 (*CmMYB12*), and Cluster-71968.7 (*CmMYB1*). Additionally, the top five correlations of *CmC3H* with *CmMYBs* included Cluster-26366.2 (*CmMYB3R*), Cluster-38227.0, Cluster-29428.0, Cluster-82527.0, and Cluster-60025.9 (*CmMYB30*) (**Figure 7**). The findings of this study suggested that these transcription factors play a crucial role in the synthesis of GCA.

### 3.8 qRT-PCR validation of selected genes

**Figure 8:**
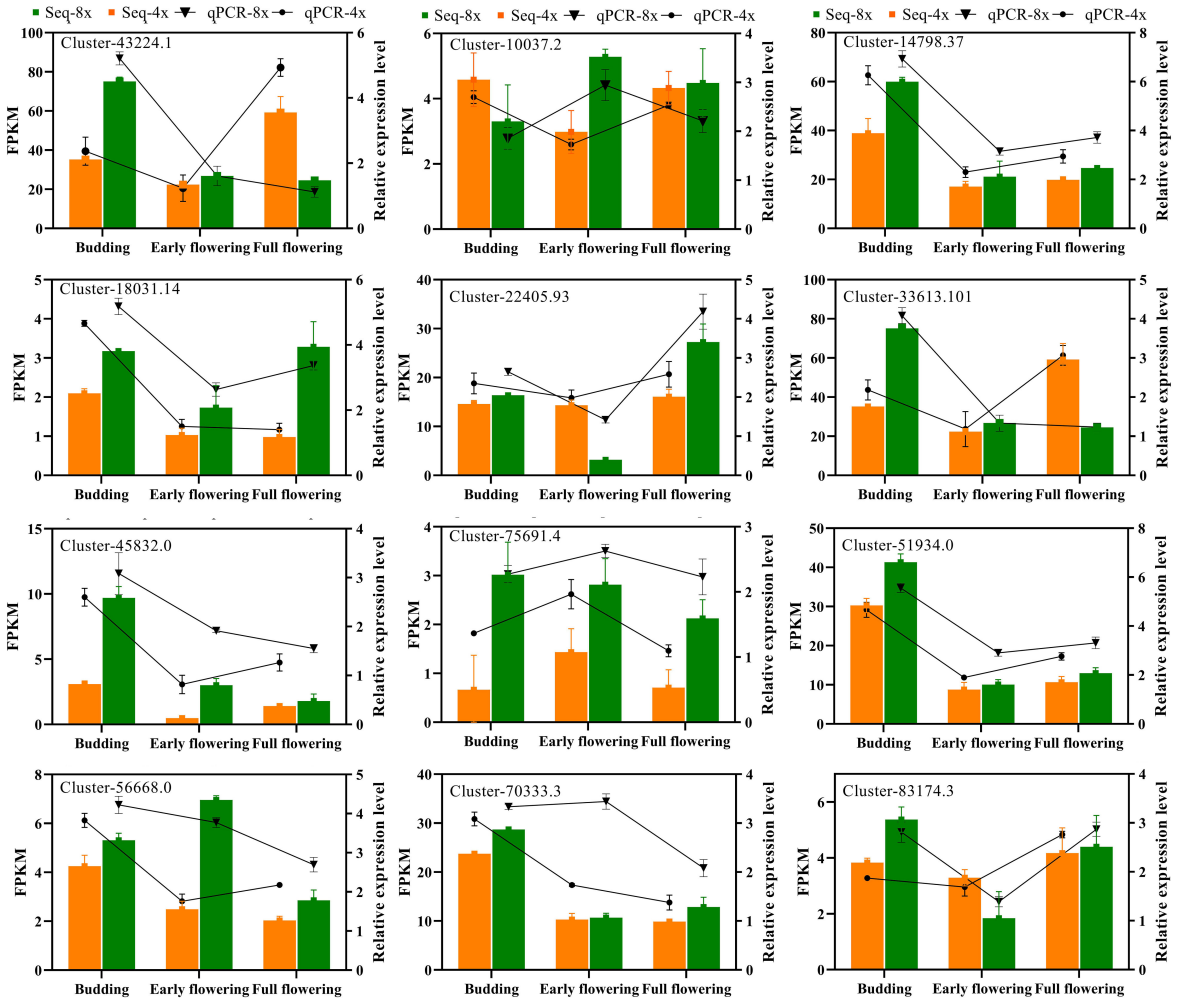
Verification of the expression patterns of RNA-seq results using qRT-PCR. The bar graphs present the results of the RNA-seq, and the line graphs present the qRT-PCR results. The scale on the right axis represents the relative expression level and the scale on the left axis represents the FPKM value. Data are means ± SD of three biological replicates. Cluster-43224.1 (Carotenoid cleavage dioxygenase), Cluster-10037.2 (LEAD-SENSITIVE I), Cluster-14798.37(Chlorophyll a-b binding protein 1D), Cluster-18031.14 (Granule-bound starch synthase 1), Cluster-33613.101 (Sucrose synthase isoform I), Cluster-22405.93 (Glutathione S-transferase DHAR2), Cluster-45832.0 (WRKY75), Cluster-51934.0 (HD-ZIP protein), Cluster-56668.0 (Flavone synthase II), Cluster-70333.3 (Soluble starch synthase I), Cluster-75691.4 (Isoflavone reductase), Cluster-83174.3 (Cold-regulated 47).

The accuracy of the transcriptome data was verified by randomly selecting 12 genes associated with carotenoid biosynthesis, ascorbate and aldarate metabolism, photosynthesis antenna proteins, starch and sucrose metabolism, and flavonoid biosynthesis for qRT-PCR validation. The relative expression levels of all the tested genes were consistent with the observed changing trend of FPKM values in the RNA-seq report during different flowering stages in tetraploid and octoploid Gongju (**Figure 8**). These findings provided compelling evidence of the robustness of the RNA-Seq report and subsequent data analysis.

## 4 Discussion

Gongju, one of the four medicinal chrysanthemums, primarily contains CGA and ICGA. CGA exhibits anti-inflammatory and antibacterial properties and is found in a variety of medicinal plants. The integration of multi-omics approaches has gained considerable traction in elucidating the synthesis of CGA compounds in medicinal plants (Hao et al., 2021; Yuan et al., 2022). The synthesis of CGA primarily relies on phenylalanine, and three pathways have been identified. HCT catalyzes the formation of *p*-coumaroyl shikimic acid from p-coumaric-CoA and shikimic acid. *p*-Coumaroyl shikimic acid is subsequently converted into CGA through HQT and *p*-coumaroyl ester 3′–hydroxylase (C3H). This pathway is prevalent in CGA biosynthesis (known as the shikimate pathway), as observed in tobacco and strawberry (Chen et al., 2020b). The second pathway for the synthesis of *p*-coumaroyl quinic acid involves the utilization of *p*-coumaric-CoA and quinic acid and the subsequent catalytic formation of CGA through the action of *p*-coumaroylester 3′-hydroxylase C3H (known as the quinine pathway). Lastly, the synthesis of CGA occurs through the catalytic action of hydroxycinnamoyl D-glucose: quinate hydroxycinnamoyl transferase (HCGQT) on the substrate consisting of caffeoyl D-glucose and quinic acid (caffeoyl glucose pathway), and the biosynthesis of CGA through this pathway is infrequently observed in plants (Wen et al., 2022). The accumulation of C3H and HQT were observed in tetraploid and octoploid Gongju flowers, and the absence of HCGQT was noted (**Figures 1B, C**). This observation suggested that CGA synthesis primarily occurs via the shikimate and quinine pathways. The exclusive synthesis CGA was observed in the quinine and shikimate pathways in pear and *Lonicera japonica* (Wen et al., 2022; Guan et al., 2023). Cinnamic acid, *p*-coumaric acid, caffeoylshikimic acid, and 4-O-*p*-coumaroylquinic acid are essential prerequisites for CGA biosynthesis. In tetraploid Gongju, *p*-coumaric acid, 4-O-*p*-coumaroylquinic acid, and CGA accumulated more extensively during the early flowering and full flowering stages than in the budding stage. Furthermore, the levels *p*-coumaric acid and 4-O-*p*-coumaroylquinic acid observed at the full flowering stage exceeded those at the early flowering stage (**Supplementary Figure S4A**). The accumulation of cinnamic acid, *p*-coumaric acid, caffeoylshikimic acid, 4-O-*p*-coumaroylquinic acid, and CGA were significantly higher during the early flowering and full flowering stages than in the budding stage in octoploid plants. Additionally, CGA and 4-O-*p*-coumaroylquinic acid content were higher at full flowering stage than at the early flowering stage (**Supplementary Figure S4B**). These study suggested that the synthesis of CGA predominantly occurs during the early flowering and full flowering stages. The levels of CGA and 4-O-*p*-coumaroylquinic acid were higher in the ctaploid plants than in the tetraploid plants during the budding, early flowering, and full flowering stages, and the budding stage showed the most pronounced difference (**Figure 1**). This observation implied that the synthesis of CGA in octoploid plants precedes or occurs at a higher rate than in tetraploid plants.

Numerous genes are involved in the biosynthesis of CGA, including *APL*, *C4H*, *4CL*, *HQT*, and *C3H*. In our study, we observed the high expression levels of *CmAPL*, *Cm4CL*, *CmHQT*, and *CmC3H* genes during the early flowering and full flowering stages compared with the budding stage in the tetraploid and octoploid plants (**Supplementary Figure S4**). The expression of *HQT* and *C3H* exhibits a positive correlation with CGA synthesis in numerous plant species (He et al., 2017; Wang et al., 2023b), and thus *HQT* and *C3H* are pivotal genes involved in the biosynthesis of CGA. The enzymatic assays demonstrated the effective catalytic activity of *MaHQT4* in the biosynthesis of CGA (Zhao et al., 2019). Heterologous overexpression of *HQT* led to a significant increase in CGA content in tobacco (Chen et al., 2023). In tetraploid and octoploid plants, the expression levels of *CmHQT* (Cluster-57865.0), and *CmC3H* (Cluster-54912.0) were considerably higher during the first and full flowering stages than in the budding stage. Therefore, these genes were utilized in screening the upstream potential transcription factor.

*MYB*, *bHLH*, and *WD40* transcription factors play pivotal roles in the regulation of CGA biosynthesis. Additionally, *AP2/ERF*, *WRKY*, and *C2H2* transcription factors have been implicated in the modulation of CGA content (Bolhassani et al., 2021; Liu et al., 2021b; Guan et al., 2023). In this study, disparity in the number of transcription factors primarily lies in the distinct combinations of FF vs BS and those of EF vs BS in tetraploid and octoploid plants. Moreover, octoploids are more abundant than their corresponding tetraploid combinations. The number of transcription factors *CmAP2/ERF*, *CmMBY*, and *CmbHLH* ranked at the top in the FF vs BS and EF vs BS combinations (**Supplementary Figure S5**). Interestingly, variations in the quantity of *CmMYB* and *CmbHLH* across different flowers stages consistently corresponded to fluctuations in CGA content. Thus, *CmMYB* and *CmbHLH* may play a crucial role in the regulation of *CmHQT* and *CmC3H* gene expression, which are well-established as rate-limiting enzymes involved in CGA synthesis.

The coexpression analysis of the MYB family with *CmC3H* (Cluster-54912.0) and *CmHQT* (Cluster-57865.0) identified 115 genes, out of which the 10 genes (Cluster-94106.0, Cluster-75874.0, Cluster-59719.0, Cluster-30519.0 and Cluster-71968.7, Cluster-26366.2, Cluster-38227.0, Cluster-29428.0, Cluster-82527.0, Cluster-60025.9) were compared with the Arabidopsis MYB family for phylogenetic evolutionary tree analysis (**Supplementary Figure S6**). The Cluster-30519.0 is closely associated with *AtMYB111/12/11* and belongs to a specific subclass. The transcription factors *AtMYB111/12/11* have been implicated in the biosynthesis of flavonoids, and the overexpression of *AtMYB11* and *AtMYB12* in transgenic plants enhances the expression of structural genes involved in the phenylpropanoid pathway and promotes the accumulation of flavonols (Luo et al., 2008; Pandey et al., 2014). The analysis of leaf and petal tissues from transgenic plants revealed that *AtMYB11* greatly enhances flavonol and CGA biosynthesis in tobacco by upregulating the expression of key biosynthetic genes (Pandey et al., 2015). Cluster-75874.0 (*CmMYB26*) and *AtMYB26/67/55/50/61* were clustered into a subclass. The overexpression of *AtMYB61* is implicated in this phenylpropanoide metabolic pathway, leading to an increase in lignin content (Newman et al., 2003). The transcription factor *AtMYB26* plays a pivotal role in the regulation of floral organ development, and the disruption of *AtMYB26* function leads to another dehiscence (Steiner-Lange et al., 2003). Cluster-94106.0 (*CmMYB5*) and *AtMYB24/21* were included in the same subclass. The expression levels of *AtMYB21* and *AtMYB24* are significantly elevated in floral organs, and they are involved in the regulation flavonol accumulation in *Arabidopsis* anthers (Zhang et al., 2021a). Cluster-71968.7 (*CmMYB1*) and *AtMYB7/4/3/32/8/6* were assigned to the same subclass. *Arabidopsis thaliana AtMYB7*, which is the closest homolog of *AtMYB4*, has been characterized as a repressor of the distinct branches of phenylpropanoid metabolism. Mutant plants lacking *atmyb4* and exhibit reduced flavonol content (Jin, 2000; Fornalé et al., 2014). In the Cluster-60025.9 (*CmMYB30*) subclass, AtMYB30/31/60 actively contribute to the development of plant cuticle, enhancing plant tolerance to drought and other types of abiotic stress (Dubos et al., 2010; Simeoni et al., 2022). Clusters Cluster-59719.0 (*CmMYB16*), Cluster-26366.2 (*CmMYB3R*), Cluster-38227.0, Cluster-29428.0, and Cluster-82527.0 did not exhibit clustering patterns with the *Arabidopsis* MYB family. A bHLH TF family member Cluster-10644.0, Cluster-11143.0, Cluster-16599.0, Cluster-34912.0, Cluster-53351.0, Cluster-32024.1 (*CmbHLH62*), Cluster-60210.0 (*CmbHLH75*), Cluster-32024.8 (*CmbHLH62*), Cluster-62341.0 (*CmbHLH75*), and Cluster-90665(*CmbHLH16*) exhibit considerably high coexpression coefficients in association with *CmCH3* and *CmHQT* (**Figure 7**). A phylogenetic tree with *Arabidopsis bHLH* family was constructed (**Supplementary Figure S7**). Cluster-32024.1 (*CmbHLH62*), Cluster-62341.0 (*CmbHLH75*), Cluster-32024.8 (*CmbHLH62*), Cluster-60210.0 (*CmbHLH75*), and *AtbHLH31/44/48/49/58/50/60* were closely associated within a specific class. The transcription factors *AtbHLH44/BEE1* (brassinosteroid-enhanced expression), *AtbHLH50*/*BEE2*, and *AtbHLH58*/*BEE3* exhibit responsiveness to brassinosteroids, ABA, and other signaling molecules and are the positive regulators of flavonoid biosynthesis (Petridis et al., 2016; Liang et al., 2023). The homology between Cluster-90665.1 (*CmbHLH16*), *AtbHLH26*, and *AtbHLH8* has been established, and the expression of *AtbHLH8* and *AtbHLH26* was induced by gibberellic acid and light signaling molecules. Furthermore, the overexpression of these genes in transgenic *A. thaliana* resulted in enhanced anthocyanin biosynthesis (Zhao et al., 2013; Wang et al., 2016). Through the fluorescence quantitative analysis of changes in the expression levels of *CmMYB12* (Cluster-30519.0), *CmMYB26* (Cluster-75874.0), *CmMYB5* (Cluster-94106.0), *CmMYB1* (Cluster-71968.7), *CmbHLH62* (Cluster-32024.1), *CmbHLH75* (Cluster-62341.0), *CmbHLH62* (Cluster-32024.8), *CmbHLH75* (Cluster-60210.0), and *CmbHLH16* (Cluster-90665.1) at different flowering stages in Gongju (**Supplementary Figure S8**), we observed that these genes exhibited consistent expression trends with *CmHQT* (Cluster-57865.0) and *CmC3H* (Cluster-54912.0) genes, further indicating their role in CGA synthesis regulation.

## 5 Conclusions

The present study employed transcriptome and metabolome analyses to investigate the CGA content between tetraploid and octoploid Gongju at differences flowers stage. The initial findings revealed that the biosynthesis of chlorogenic acid in Gongju encompasses both the shikimate and quinine pathways. The higher chlorogenic acid content in octoploid Gongju compared to tetraploid Gongju can be attributed primarily to the elevated expression levels of *CmHQT* (Cluster-37095.3 and Cluster-37095.8) and *CmC3H* (Cluster-67171.0) genes. Furthermore, co-expression analysis revealed the involvement of transcription factors such as *CmMYB* (Cluster-30519.0, Cluster-75874.0, Cluster-94106.0, Cluster-71968.7) and *CmbHLH* (Cluster-32024.1, Cluster-62341.0, Cluster-32024.8, Cluster-62341.0, cluster-90665.1) in the regulation of chlorogenic acid synthesis between octoploid and tetraploid Gongju. Meanwhile, the verification of whether these transcription factors can bind to the structural genes *CmHQT* and *CmC3H* still necessitates further investigation.

## Data Availability

The original contributions presented in the study are publicly available. This data can be found here: National Center for Biotechnology Information (NCBI) BioProject database under accession number PRJNA1154322. The raw metabolome data are stored in the MetaLights database (MTBLS11095).

## Supplementary material

Supplementary Figure 1 Overview and verification of RNA-seq data. **(A)** Analysis of sample correlation. **(B)** Principal Component Analysis (PCA) based on the dataset of fragments per kilobaseexon per million reads mapped. 4SB, 4EF and 4FF represent the bud stage, early flowering stage, full flowering stage of tetraploid, respectively; 8SB, 8EF, and 8FF correspond to the bud stage, early flowering stage, and full flowering stage of octoploid, respectively.

Supplementary Figure 2 Metabolomic data principal component analysis. Budding stage (BS), early flowering stage (EF) and full flowering stage (FF). 4 and 8: tetraploid and octoploid.

Supplementary Figure 3 Clustering dendrogram of DEGs, with dissimilarity based on the topological overlap, together with assigned module colors. **(A)** *CmC3H* and *CmHQT* with *CmMYBs*. **(B)** *CmC3H* and *CmHQT* with *CmbHLHs*.

Supplementary Figure 4 DEGs and DAMs in CGA biosynthesis pathways were compared at different flowering stages of tetraploids **(A)**, and octopoids **(B)**, respectively. APL: Cluster-39849.10, Cluster-39849.7, Cluster-39849.6, Cluster-39849.1; Cluster-39849.0; C4H: Cluster-52575.0, Cluster-52575.1; 4CL: Cluster-71418.4, Cluster-58578.4, Cluster-77720.0, Cluster-35536.4, Cluster-93180.0, Cluster-33436.59; HQT: Cluster-57865.0, Cluster-81585.0, Cluster-37095.0, Cluster-37095.5, Cluster-70993.0; C3H: Cluster-67171.0, Cluster-54912.0; MWS20194: Cinnamic acid; pme1439: p-Coumaric acid; Lmfp101801: Caffeoylshikimic acid; pma6460: 4-O-p-Coumaroylquinic acid; mws0178: Chlorogenic acid.

Supplementary Figure 5 Number of differential expression of transcription factors.

Supplementary Figure 6 Unrooted phylogenetic tree of MYB between Gongju and *A. thaliana.* Gongju Cluster-94106.0 (*CmMYB5*), Cluster-75874.0 (*CmMYB26*), Cluster-59719.0 (*CmMYB16*), Cluster-30519.0 (*CmMYB12*) and Cluster-71968.7 (*CmMYB1*), Cluster-26366.2 (*CmMYB3R*), Cluster-38227.0, Cluster-29428.0, Cluster-82527.0, Cluster-60025.9); Arabidopsis thaliana (Tair).

Supplementary Figure 7 Unrooted phylogenetic tree of bHLH between Gongju and *A. thaliana.* Gongju bHLHs Cluster-10644.0, Cluster-11143.0, Cluster-16599.0, Cluster-34912.0, Cluster-53351.0, Cluster-32024.1 (*CmbHLH62*), Cluster-60210.0 (*CmbHLH75*), Cluster-32024.8, Cluster-62341.0, and Cluster-90665 (*CmbHLH16*)); Arabidopsis thaliana (Tair).

Supplementary Figure 8 Analysis of differentially expressed genes by qPCR. Cluster-90665.1(*CmbHLH16*), Cluster-62341.0 (*CmbHLH75*), Cluster-32024.8 (*CmbHLH62*), Cluster-32024.1 (*CmbHLH62*), Cluster-71968.7 (*CmMYB1*), Cluster-94106.0 (*CmMYB5*), Cluster-75874.0 (*CmMYB26*), Cluster-30519.0 (*CmMYB12*), Cluster-54912.0 (*CmC3H*), Cluster-57865.0 (*CmHQT*).
