# Supplementary figures and images for: Transcriptome and metabolome profiling unveil the accumulation of chlorogenic acid in autooctoploid Gongju
Source: Front Plant Sci. 2024 Nov 1;15:1461357. doi: 10.3389/fpls.2024.1461357 (PMC11563975; doi:10.3389/fpls.2024.1461357)

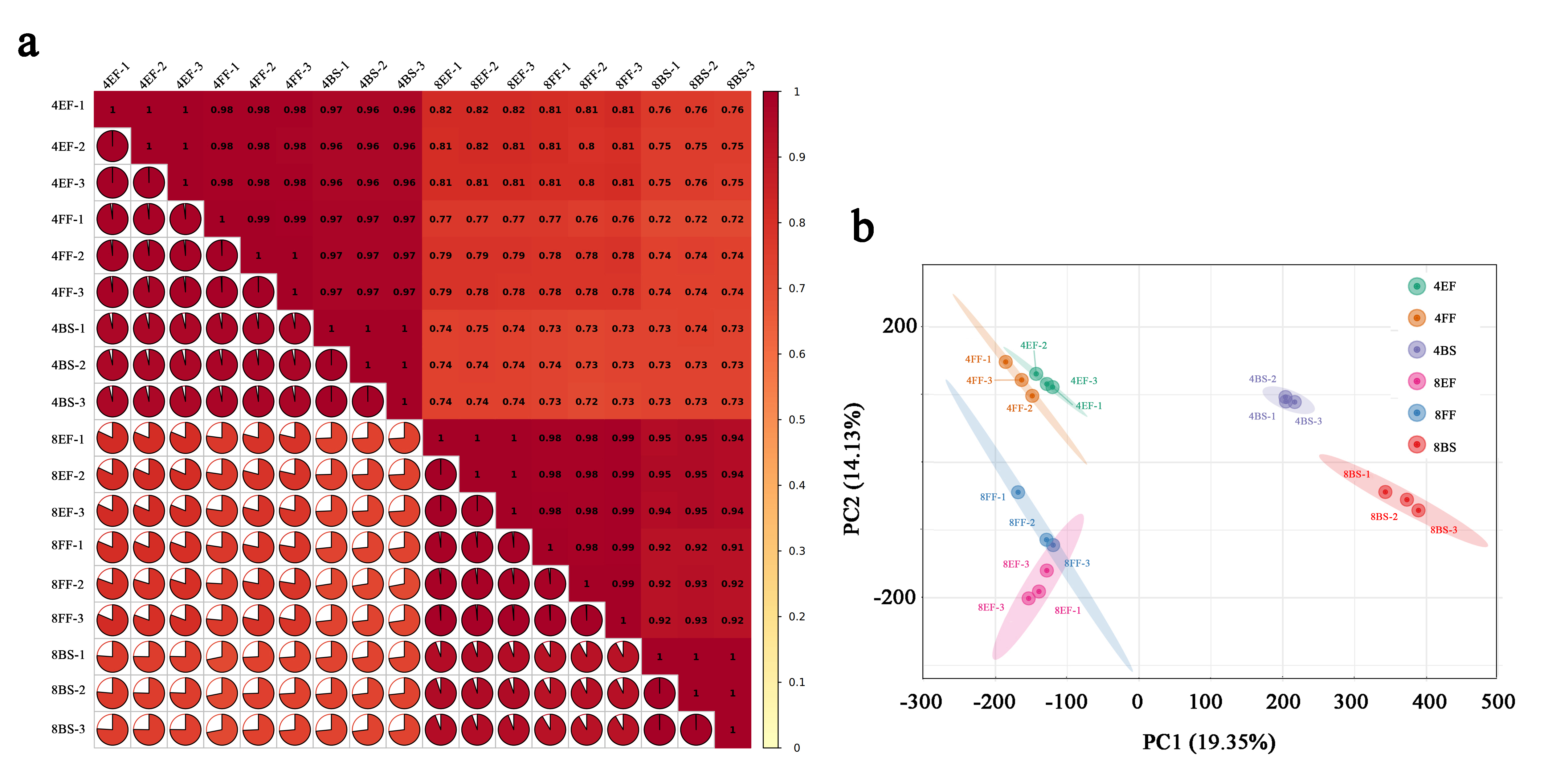

Supplement: Supplementary Figure 1 — Overview and verification of RNA-seq data. (A) Analysis of sample correlation. (B) Principal Component Analysis (PCA) based on the dataset of fragments per kilobaseexon per million reads mapped. 4SB, 4EF and 4FF represent the bud stage, early flowering stage, full flowering stage of tetraploid, respectively; 8SB, 8EF, and 8FF correspond to the bud stage, early flowering stage, and full flowering stage of octoploid, respectively. [file Image1.jpeg]

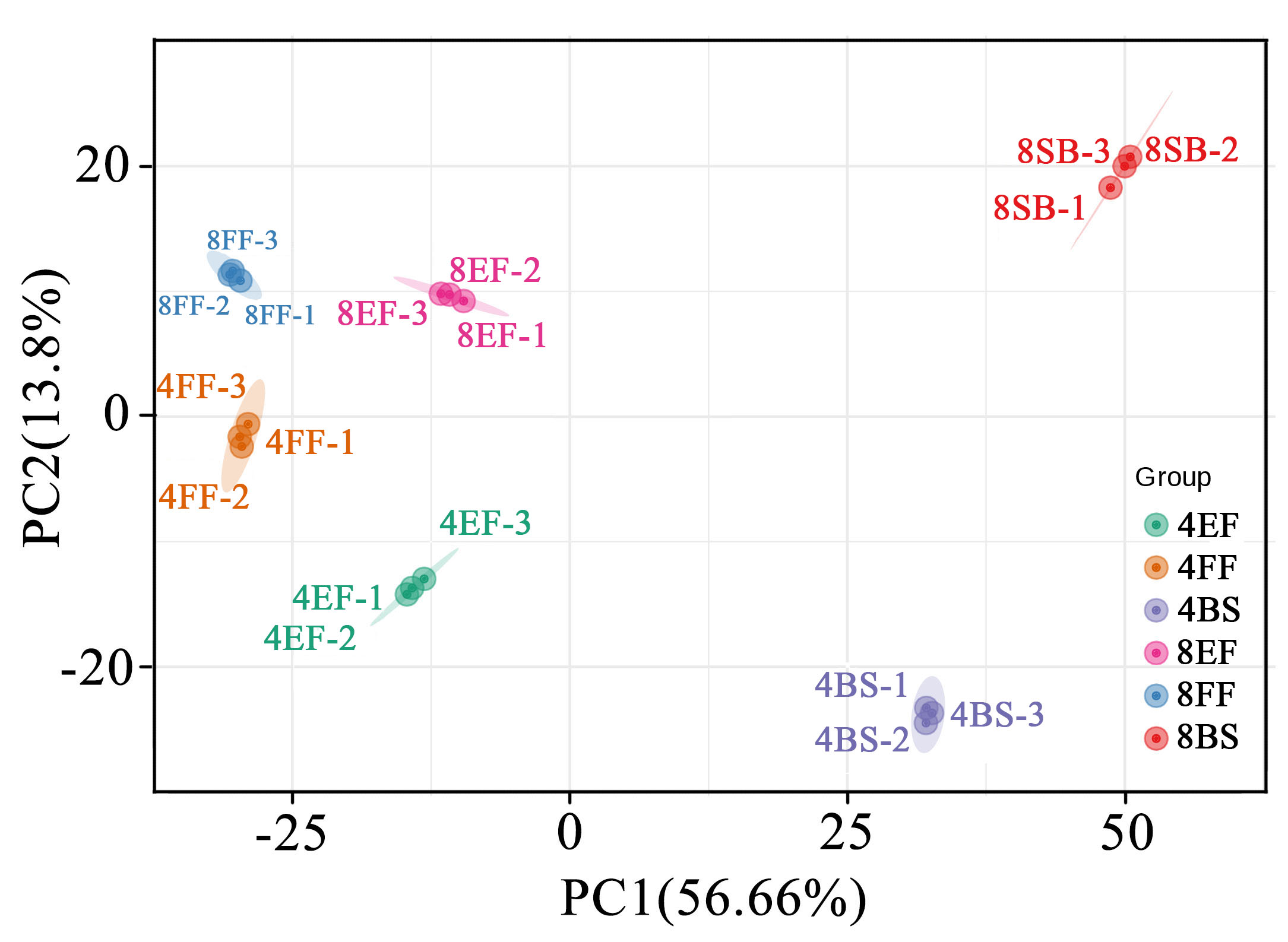

Supplement: Supplementary Figure 2 — Metabolomic data principal component analysis. Budding stage (BS), early flowering stage (EF) and full flowering stage (FF). 4 and 8: tetraploid and octoploid. [file Image2.jpeg]

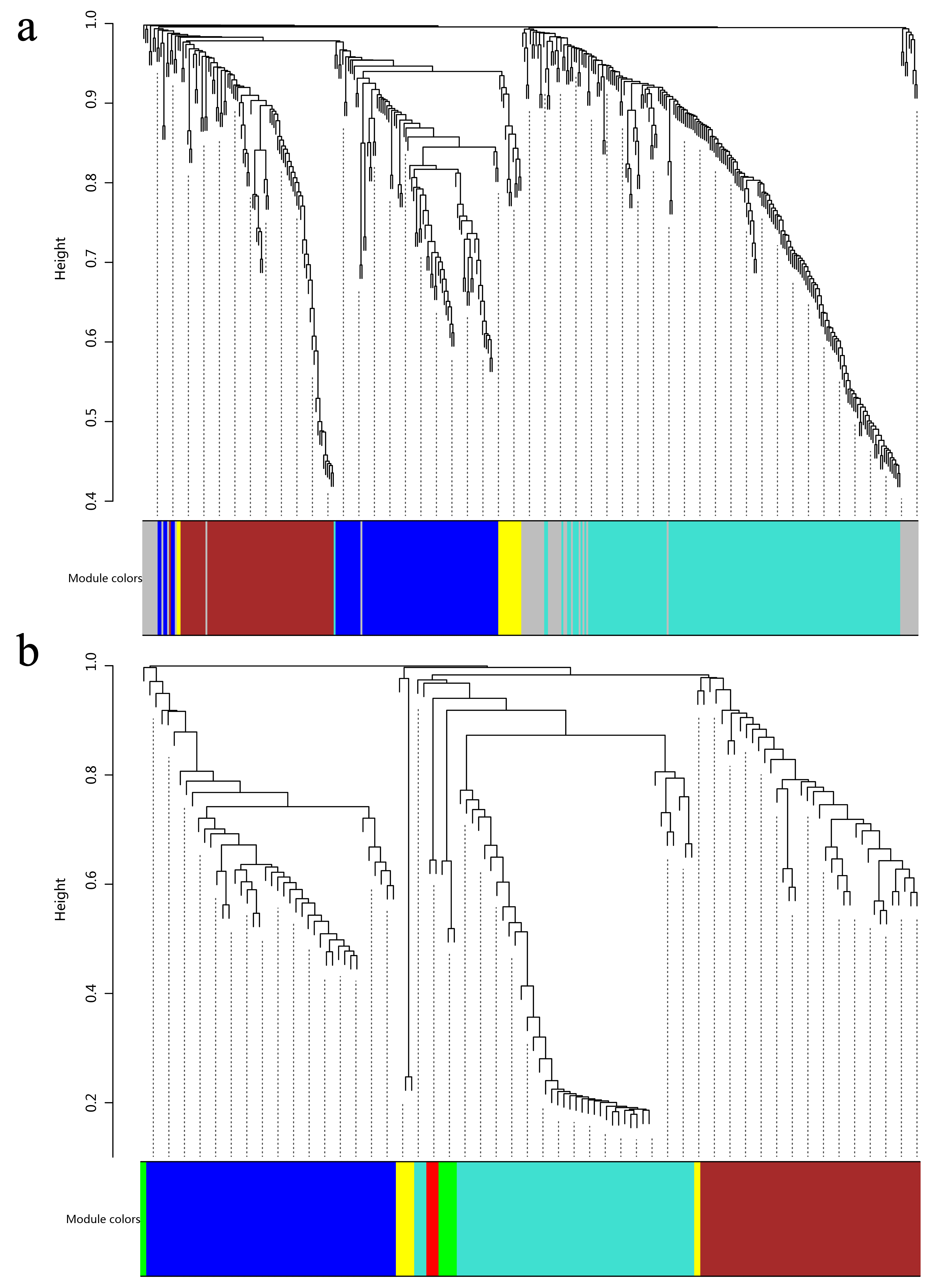

Supplement: Supplementary Figure 3 — Clustering dendrogram of DEGs, with dissimilarity based on the topological overlap, together with assigned module colors. (A) CmC3H and CmHQT with CmMYBs. (B) CmC3H and CmHQT with CmbHLHs. [file Image3.jpeg]

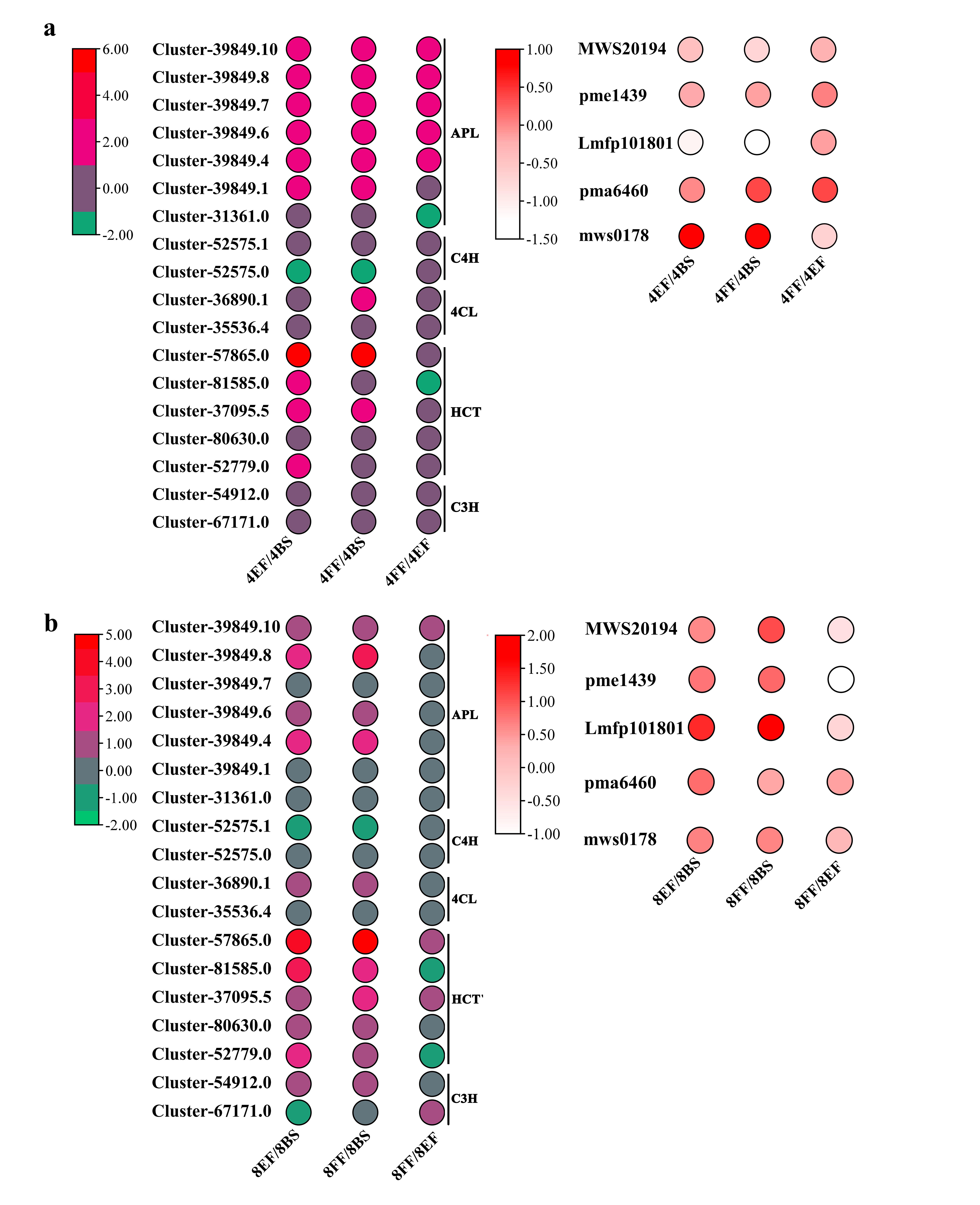

Supplement: Supplementary Figure 4 — DEGs and DAMs in CGA biosynthesis pathways were compared at different flowering stages of tetraploids (A), and octopoids (B), respectively. APL: Cluster-39849.10, Cluster-39849.7, Cluster-39849.6, Cluster-39849.1; Cluster-39849.0; C4H: Cluster-52575.0, Cluster-52575.1; 4CL: Cluster-71418.4, Cluster-58578.4, Cluster-77720.0, Cluster-35536.4, Cluster-93180.0, Cluster-33436.59; HQT: Cluster-57865.0, Cluster-81585.0, Cluster-37095.0, Cluster-37095.5, Cluster-70993.0; C3H: Cluster-67171.0, Cluster-54912.0; MWS20194: Cinnamic acid; pme1439: p-Coumaric acid; Lmfp101801: Caffeoylshikimic acid; pma6460: 4-O-p-Coumaroylquinic acid; mws0178: Chlorogenic acid. [file Image4.jpeg]

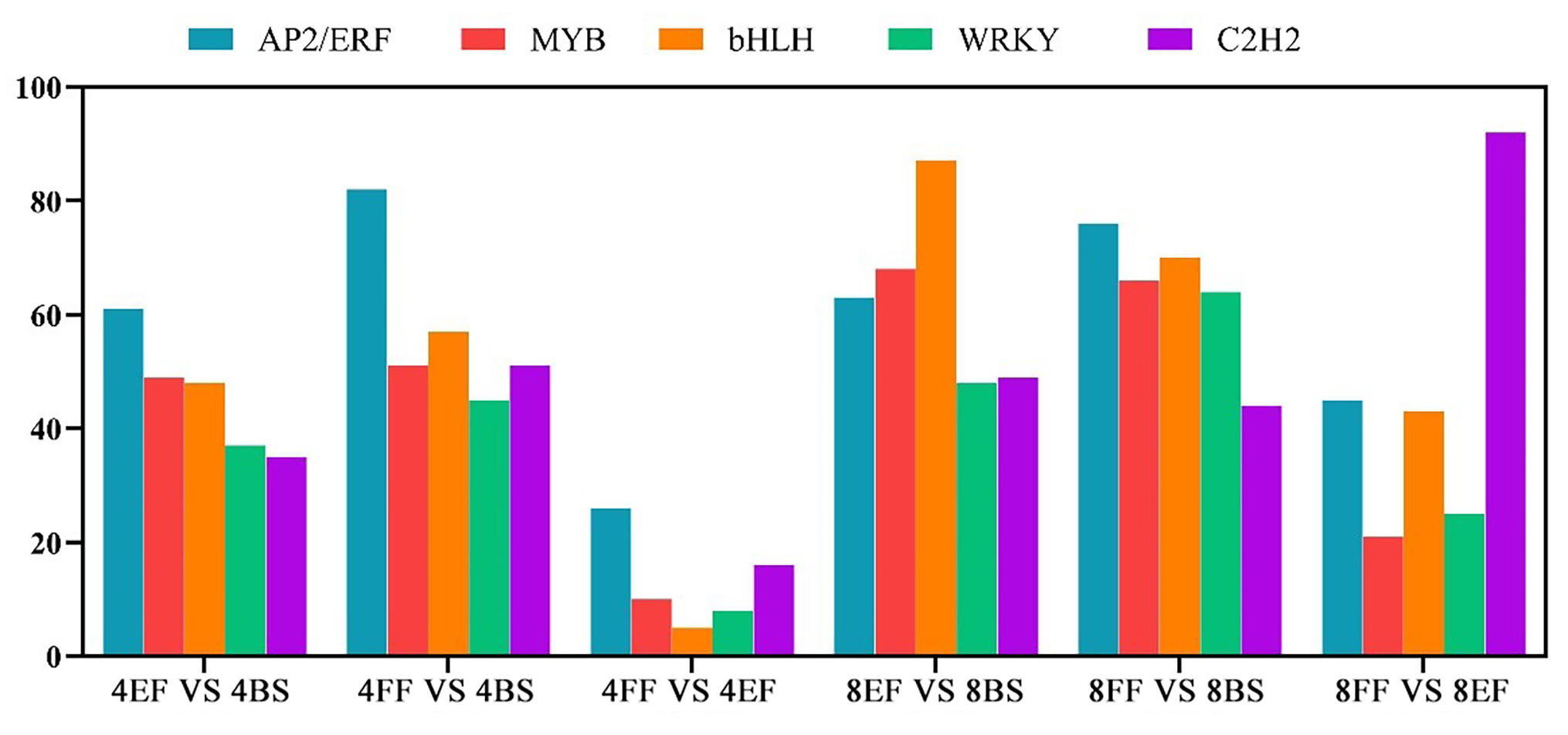

Supplement: Supplementary Figure 5 — Number of differential expression of transcription factors. [file Image5.jpeg]

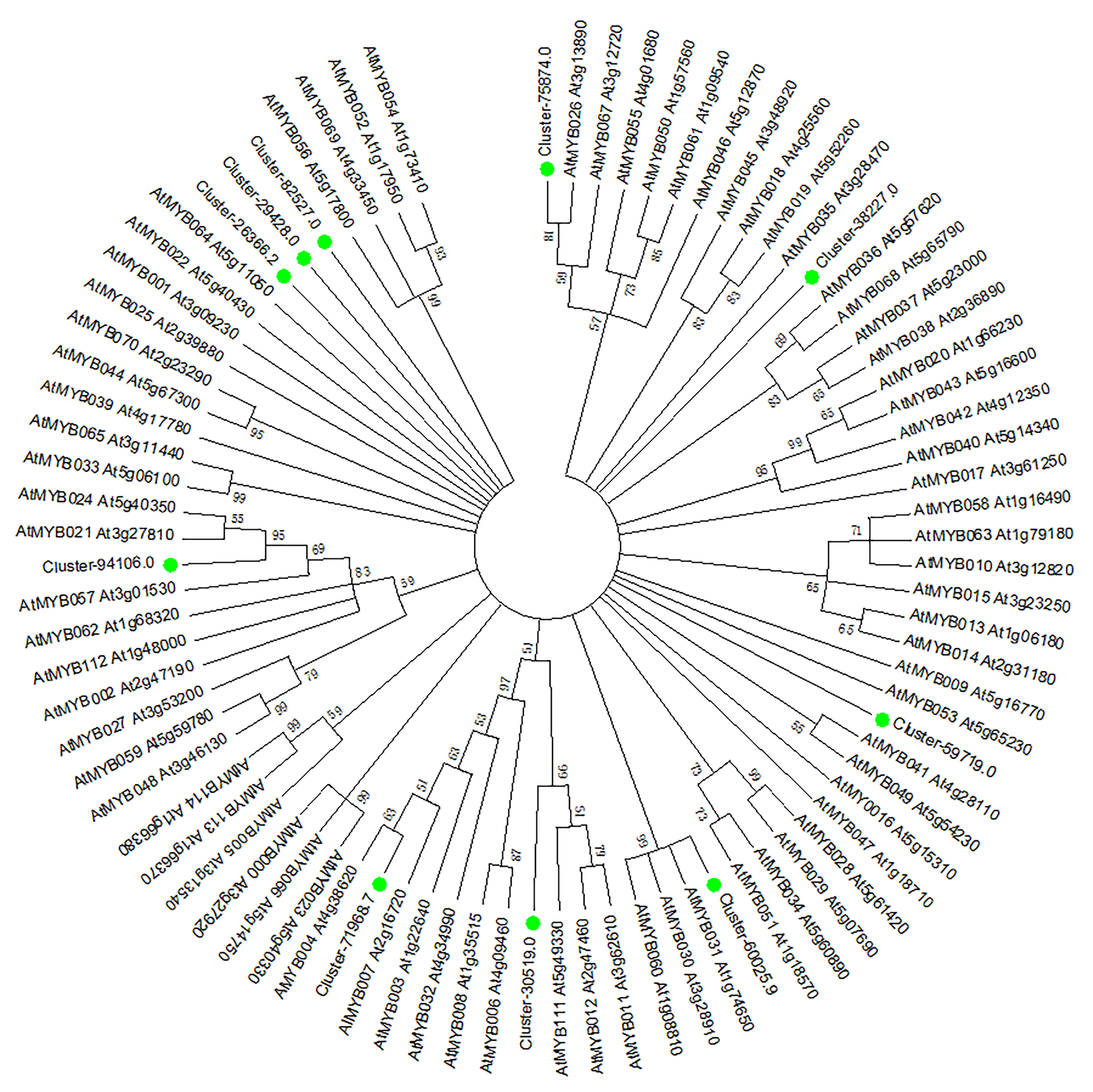

Supplement: Supplementary Figure 6 — Unrooted phylogenetic tree of MYB between Gongju and A. thaliana. Gongju Cluster-94106.0 (CmMYB5), Cluster-75874.0 (CmMYB26), Cluster-59719.0 (CmMYB16), Cluster-30519.0 (CmMYB12) and Cluster-71968.7 (CmMYB1), Cluster-26366.2 (CmMYB3R), Cluster-38227.0, Cluster-29428.0, Cluster-82527.0, Cluster-60025.9); Arabidopsis thaliana (Tair). [file Image6.jpeg]

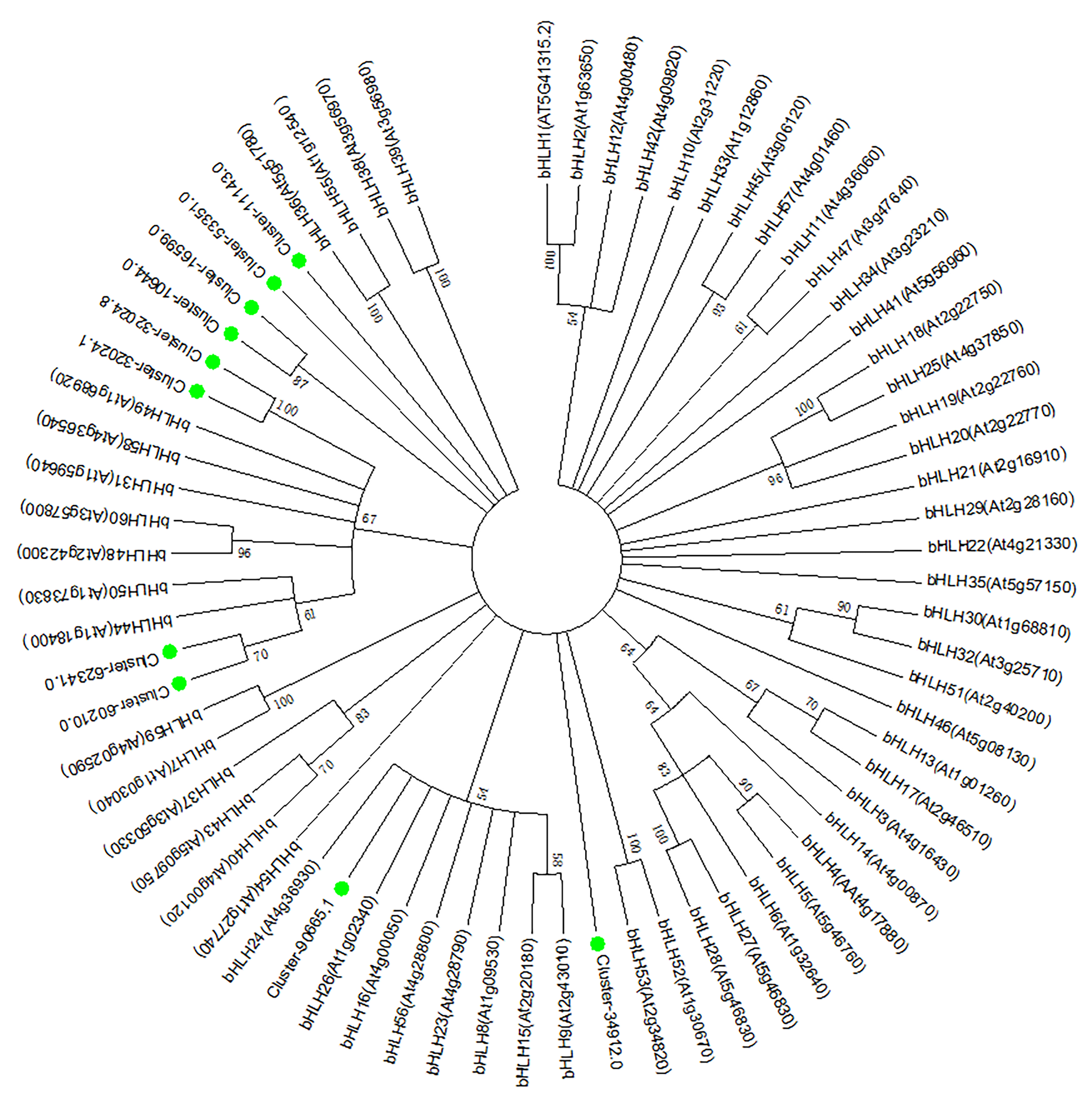

Supplement: Supplementary Figure 7 — Unrooted phylogenetic tree of bHLH between Gongju and A. thaliana. Gongju bHLHs Cluster-10644.0, Cluster-11143.0, Cluster-16599.0, Cluster-34912.0, Cluster-53351.0, Cluster-32024.1 (CmbHLH62), Cluster-60210.0 (CmbHLH75), Cluster-32024.8, Cluster-62341.0, and Cluster-90665 (CmbHLH16)); Arabidopsis thaliana (Tair). [file Image7.jpeg]

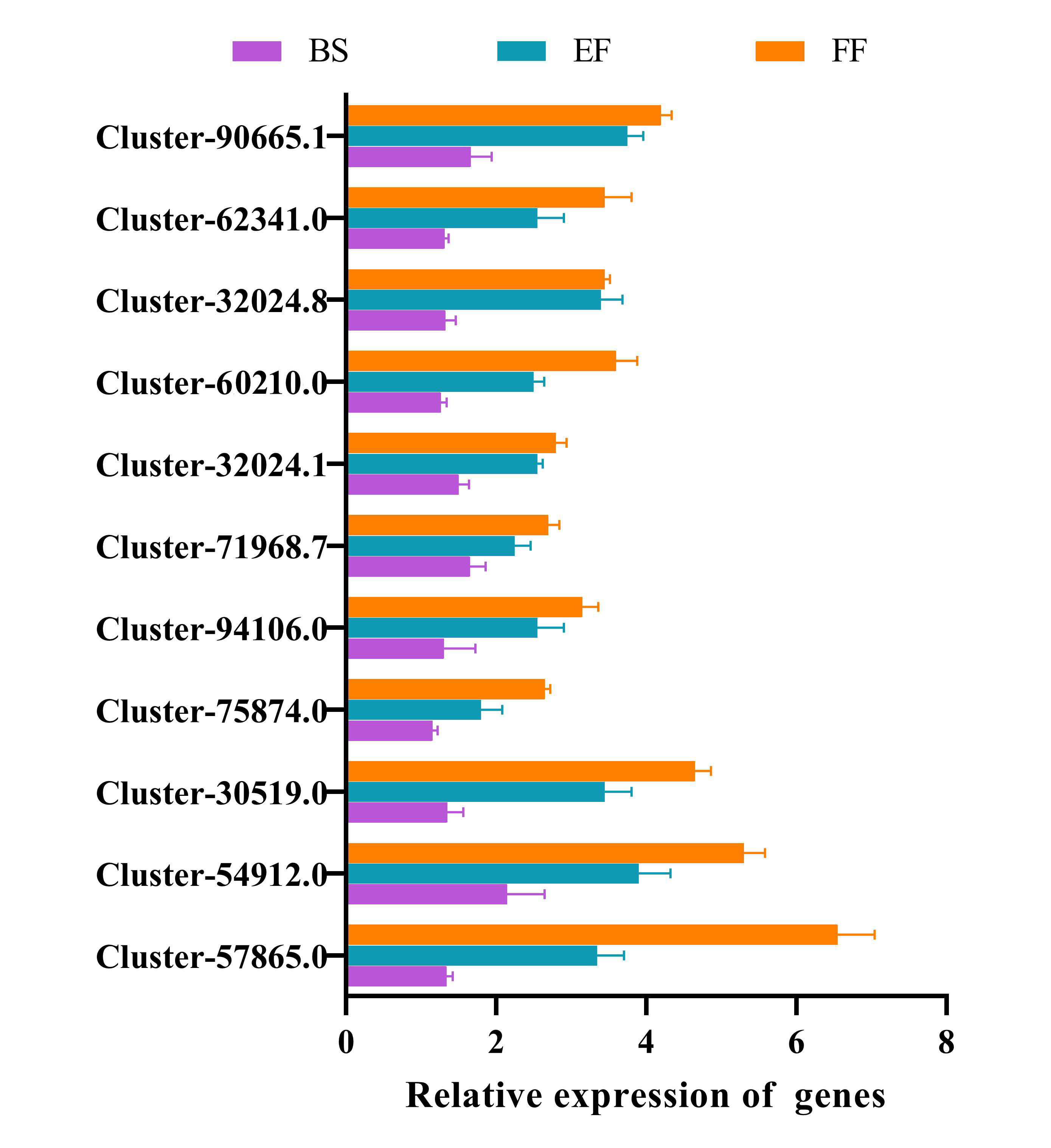

Supplement: Supplementary Figure 8 — Analysis of differentially expressed genes by qPCR. Cluster-90665.1(CmbHLH16), Cluster-62341.0 (CmbHLH75), Cluster-32024.8 (CmbHLH62), Cluster-32024.1 (CmbHLH62), Cluster-71968.7 (CmMYB1), Cluster-94106.0 (CmMYB5), Cluster-75874.0 (CmMYB26), Cluster-30519.0 (CmMYB12), Cluster-54912.0 (CmC3H), Cluster-57865.0 (CmHQT). [file Image8.jpeg]
