# Supplementary material for: Transcriptome and metabolome profiling unveil the accumulation of chlorogenic acid in autooctoploid Gongju
Source: Front Plant Sci. 2024 Nov 1;15:1461357. doi: 10.3389/fpls.2024.1461357 (PMC11563975; doi:10.3389/fpls.2024.1461357)
Supplement: Supplementary file 9 [file Table1.docx]

**Table S1 Primer names and sequences used in this study**

| Prime name | Sequence (5' to 3') |
| --- | --- |
| Cluster-43224.1-F | TATAGTAGCTAGTCGTATGT |
| Cluster-43224.1-R | TAGCTTGTTAATGTCACT |
| Cluster-22405.93-F | AAGCTGTACCACCTTGAG |
| Cluster-22405.93-R | ACCCAGCAATGACATATTC |
| Cluster-14798.37-F | GTTACAGAATTGCTGGTGG |
| Cluster-14798.37-R | GCAAGGTGGTCAGCCAAG |
| Cluster-18031.14-F | ATTCAGGGAATCTTCAATG |
| Cluster-18031.14- R | GTAACTTACAAGAACTAT |
| Cluster-70333.3-F | GTTGGATGCATTATGGATTGC |
| Cluster-70333.3-R | GCTGACGTAAGGTGGATC |
| Cluster-33613.101-F | ACCTAAGCACATCTGATA |
| Cluster-33613.101-R | GCTCTCATGTCTCAAAGAC |
| Cluster-10037.2-F | GCTCCTTGGAAGCCCAT |
| Cluster-10037.2-R | TGCATGATGTGCAACCATCA |
| Cluster-56668.0-F | AGAAGGCGATGTTCTAA |
| Cluster-56668.0-R | ATCCTCTGACCTTGGAGC |
| ClEF1α-F | TTTTGGTATCTGGTCCTGGAG |
| ClEF1α-R | CCATTCAAGCGACAGACTCA |
| Cluster-75691.4-F | GtATCTTCGACCAGATGA |
| Cluster-75691.4-R | TGCCATTGCATCACTCA |
| Cluster-45832.0-F | ATAGATGGAGGAAGTATG |
| Cluster-45832.0-R | GCTAGCAGAAGAATAGA |
| Cluster-51934.0-F | AGAGAGGTTGAGAGACTC |
| Cluster-51934.0-R | ATCATGACCATTGGACTT |
| Cluster-83174.3-F | CTCCTCCGTCTTCTTGTGA |
| Cluster-83174.3-R | CAGTCTAGTGATGAGGA |
| Cluster-30519.0-F | AAGAAGAATGCATGTAA |
| Cluster-30519.0-R | CATACCATCATCACCACCATA |
| Cluster-75874.0-F | ATTATGACCTCATCCTTG |
| Cluster-75874.0-R | CATTATAGTACTTACCTTG |
| Cluster-94106.0-F | GCTAGATCAGCAGGTC |
| Cluster-94106.0-R | GTCAGATAGATAGTGATT |
| Cluster-32024.1-F | TGATGCCTCATCACAACCGT |
| Cluster-32024.1-R | TCATATGACCATGGAATC |
| Cluster-71968.7-F | ACTAAGCTCAATATCACCTCA |
| Cluster-71968.7-R | TGTACCATTAGTACTACAC |
| Cluster-62341.0-F | CAGGATGTTATAAGACAAT |
| Cluster-62341.0-R | CTGTAGAAGGTCTCCATAC |
| Cluster-32024.8-F | ATAGCATTGTTCCTGATGTATC |
| Cluster-32024.8-R | GCTGATCCTGTCCTAATGCCA |
| Cluster-62341.0-F | CCAGGATGTTATAAGACAAT |
| Cluster-62341.0-R | GTCTCCATACCCTTCTGCA T |
| Cluster-90665.1-F | TGATGCCATTAGCTATGCAAC |
| Cluster-90665.1-R | CATTGCAGCCATTCGACTATA |
| Cluster-57865.0-F | TGCAAAGAGATAGAAGAATG |
| Cluster-57865.0-R | ACTTCATTGTCATTGTCTGC |
| Cluster-54912.0-F | ACGACTATCGGCTACTACCT |
| Cluster-54912.0-R | CATGTATGTCACCATCCCT |
