# Supplementary material for: Transcriptome and metabolome profiling unveil the accumulation of chlorogenic acid in autooctoploid Gongju
Source: Front Plant Sci. 2024 Nov 1;15:1461357. doi: 10.3389/fpls.2024.1461357 (PMC11563975; doi:10.3389/fpls.2024.1461357)
Supplement: Supplementary file 10 [file Table2.docx]

| **Sample** | **Raw Reads** | **Clean Reads** | **Clean Base(G)** | **Error Rate(%)** | **Q20**  **(%)** | **Q30**  **(%)** | **GC Content(%)** |
| --- | --- | --- | --- | --- | --- | --- | --- |
| 4BS -1 | 86834200 | 82580250 | 12.39 | 0.04 | 96.86 | 90.53 | 44.39 |
| 4BS -2 | 69912040 | 67430146 | 10.11 | 0.04 | 96.75 | 90.18 | 44.42 |
| 4BS -3 | 79035792 | 72262448 | 10.84 | 0.04 | 96.93 | 90.73 | 44.32 |
| 4EF-1 | 79720358 | 76555762 | 11.48 | 0.04 | 96.69 | 90.02 | 44.61 |
| 4EF -2 | 83723122 | 78577794 | 11.79 | 0.04 | 96.92 | 90.71 | 44.72 |
| 4EF -3 | 80289398 | 76631330 | 11.49 | 0.04 | 96.9 | 90.65 | 44.63 |
| 4FF-1 | 77782104 | 74803172 | 11.22 | 0.04 | 96.69 | 90.03 | 44.84 |
| 4FF-2 | 84834238 | 80764306 | 12.11 | 0.04 | 96.66 | 89.95 | 44.63 |
| 4FF-3 | 76860200 | 72687688 | 10.9 | 0.04 | 96.93 | 90.76 | 44.73 |
| 8BS -1 | 84701556 | 80036580 | 12.01 | 0.04 | 96.93 | 90.73 | 44.19 |
| 8BS -2 | 62633594 | 59619324 | 8.94 | 0.04 | 96.87 | 90.53 | 44.09 |
| 8BS -3 | 75648048 | 71938370 | 10.79 | 0.04 | 96.51 | 89.73 | 44.07 |
| 8EF -1 | 77143318 | 73369212 | 11.01 | 0.04 | 96.92 | 90.71 | 43.94 |
| 8EF -2 | 81370630 | 74514664 | 11.18 | 0.04 | 97 | 90.96 | 44.13 |
| 8EF -3 | 67763316 | 64335106 | 9.65 | 0.04 | 96.57 | 89.88 | 44.07 |
| 8FF-1 | 77607566 | 73989866 | 11.1 | 0.04 | 96.86 | 90.56 | 44.68 |
| 8FF-2 | 89466452 | 84246678 | 12.64 | 0.04 | 96.85 | 90.53 | 44.35 |
| 8FF-3 | 71092910 | 67903806 | 10.19 | 0.04 | 96.31 | 89.16 | 44.18 |

**Table S2 Comprehensive overview of transcriptomic data**

Budding stage (BS), early flowering stage (EF) and full flowering stage (FF)
