# Supplementary material for: Transcriptome and metabolome profiling unveil the accumulation of chlorogenic acid in autooctoploid Gongju
Source: Front Plant Sci. 2024 Nov 1;15:1461357. doi: 10.3389/fpls.2024.1461357 (PMC11563975; doi:10.3389/fpls.2024.1461357)
Supplement: Supplementary file 13 [file Table5.docx]

**Table S5 KEGG classifications of differentially expressed genes in same flowering stage of tetraploid and octoploid Gongju**

| **Number** | **Group** | **KEGG ID** | **Description** | **Rich Factor** | ***p* Value** | **Gene Number** | | |
| --- | --- | --- | --- | --- | --- | --- | --- | --- |
|  |  |  |  |  |  | **Total** | **Up** | **Down** |
| 1 | **4BS/8BS** | ko00940 | Phenylpropanoid biosynthesis | 5.01% | 3.61E-19 | 69 | 65 | 4 |
| 2 |  | ko01040 | Biosynthesis of unsaturated fatty acids | 2.91% | 8.44E-18 | 40 | 38 | 2 |
| 3 |  | ko00941 | Flavonoid biosynthesis | 1.89% | 9.66E-10 | 26 | 26 | 0 |
| 4 |  | ko01212 | Fatty acid metabolism | 4.14% | 2.74E-09 | 57 | 49 | 8 |
| 5 |  | ko00520 | Amino sugar and nucleotide sugar metabolism | 4.72% | 6.89E-07 | 65 | 49 | 16 |
| 6 |  | ko04141 | Protein processing in endoplasmic reticulum | 6.9% | 4.69E-05 | 95 | 70 | 25 |
| 7 |  | ko00945 | Stilbenoid, diarylheptanoid and gingerol biosynthesis | 0.87% | 0.000572 | 12 | 12 | 0 |
| 8 |  | ko00966 | Glucosinolate biosynthesis | 0.51% | 0.00078 | 7 | 1 | 6 |
| 9 |  | ko00943 | Isoflavonoid biosynthesis | 0.58% | 0.000896 | 8 | 8 | 0 |
| 10 |  | ko00942 | Anthocyanin biosynthesis | 0.58% | 0.001051 | 8 | 7 | 1 |
| 11 |  | ko00380 | Tryptophan metabolism | 1.82% | 0.001446 | 25 | 16 | 9 |
| 12 |  | ko00592 | alpha-Linolenic acid metabolism | 1.67% | 0.001829 | 23 | 20 | 3 |
| 13 |  | ko00073 | Cutin, suberine and wax biosynthesis | 1.09% | 0.00195 | 15 | 15 | 0 |
| 14 |  | ko04626 | Plant-pathogen interaction | 12.94% | 0.003268 | 178 | 135 | 43 |
| 15 |  | ko04016 | MAPK signaling pathway - plant | 4.87% | 0.00383 | 67 | 41 | 26 |
| 16 |  | ko03250 | Viral life cycle - HIV-1 | 1.24% | 0.004643 | 17 | 4 | 13 |
| 17 |  | ko00901 | Indole alkaloid biosynthesis | 0.36% | 0.006199 | 5 | 5 | 0 |
| 18 |  | ko04075 | Plant hormone signal transduction | 6.83% | 0.007435 | 94 | 71 | 23 |
| 19 |  | ko00905 | Brassinosteroid biosynthesis | 0.51% | 0.011481 | 7 | 7 | 0 |
| 20 |  | ko00563 | Glycosylphosphatidylinositol (GPI)-anchor biosynthesis | 0.94% | 0.024133 | 13 | 1 | 12 |
| 21 |  | ko00910 | Nitrogen metabolism | 0.73% | 0.025216 | 10 | 7 | 3 |
| 22 |  | ko00601 | Glycosphingolipid biosynthesis - lacto and neolacto series | 0.15% | 0.028336 | 2 | 2 | 0 |
| 23 |  | ko00902 | Monoterpenoid biosynthesis | 0.58% | 0.028475 | 8 | 8 | 0 |
| 24 |  | ko00908 | Zeatin biosynthesis | 0.94% | 0.033672 | 13 | 11 | 2 |
| 25 |  | ko03010 | Ribosome | 5.38% | 0.044447 | 74 | 19 | 55 |
| 26 |  | ko01110 | Biosynthesis of secondary metabolites | 5.87% | 0.044783 | 356 | 297 | 59 |
| 27 |  | ko00071 | Fatty acid degradation | 1.74% | 0.049832 | 24 | 23 | 1 |
| 1 | **4EF/8EF** | ko01212 | Fatty acid metabolism | 3.69% | 0.000 | 138 | 132 | 6 |
| 2 |  | ko01040 | Biosynthesis of unsaturated fatty acids | 1.68% | 0.000 | 63 | 63 | 0 |
| 3 |  | ko01100 | Metabolic pathways | 45.79% | 0.000 | 1714 | 1498 | 216 |
| 4 |  | ko04141 | Protein processing in endoplasmic reticulum | 6.65% | 0.000 | 249 | 240 | 9 |
| 5 |  | ko00062 | Fatty acid elongation | 1.02% | 0.000 | 38 | 36 | 2 |
| 6 |  | ko00520 | Amino sugar and nucleotide sugar metabolism | 3.69% | 0.000 | 138 | 113 | 25 |
| 7 |  | ko03250 | Viral life cycle - HIV-1 | 1.23% | 0.000 | 46 | 44 | 2 |
| 8 |  | ko00514 | Other types of O-glycan biosynthesis | 0.51% | 0.000 | 19 | 19 | 0 |
| 9 |  | ko04145 | Phagosome | 2.24% | 0.000 | 84 | 80 | 4 |
| 10 |  | ko00943 | Isoflavonoid biosynthesis | 0.43% | 0.000 | 16 | 16 | 0 |
| 11 |  | ko00061 | Fatty acid biosynthesis | 1.63% | 0.000 | 61 | 56 | 5 |
| 12 |  | ko00073 | Cutin, suberine and wax biosynthesis | 0.91% | 0.000 | 34 | 30 | 4 |
| 13 |  | ko03010 | Ribosome | 5.61% | 0.000 | 210 | 205 | 5 |
| 14 |  | ko04070 | Phosphatidylinositol signaling system | 2.03% | 0.000 | 76 | 66 | 10 |
| 15 |  | ko00604 | Glycosphingolipid biosynthesis - ganglio series | 0.67% | 0.000 | 25 | 14 | 11 |
| 16 |  | ko00330 | Arginine and proline metabolism | 1.58% | 0.000 | 59 | 53 | 6 |
| 17 |  | ko00190 | Oxidative phosphorylation | 2.75% | 0.000 | 103 | 97 | 6 |
| 18 |  | ko03040 | Spliceosome | 6.28% | 0.000 | 235 | 215 | 20 |
| 19 |  | ko00280 | Valine, leucine and isoleucine degradation | 1.76% | 0.000 | 66 | 51 | 15 |
| 20 |  | ko00561 | Glycerolipid metabolism | 2.3% | 0.000 | 86 | 77 | 9 |
| 21 |  | ko03008 | Ribosome biogenesis in eukaryotes | 2.35% | 0.001 | 88 | 80 | 8 |
| 22 |  | ko00515 | Mannose type O-glycan biosynthesis | 0.13% | 0.001 | 5 | 5 | 0 |
| 23 |  | ko00040 | Pentose and glucuronate interconversions | 2.19% | 0.001 | 82 | 66 | 16 |
| 24 |  | ko00053 | Ascorbate and aldarate metabolism | 1.84% | 0.002 | 69 | 58 | 11 |
| 25 |  | ko00600 | Sphingolipid metabolism | 1.23% | 0.002 | 46 | 32 | 14 |
| 26 |  | ko00650 | Butanoate metabolism | 0.59% | 0.002 | 22 | 20 | 2 |
| 27 |  | ko00250 | Alanine, aspartate and glutamate metabolism | 1.2% | 0.003 | 45 | 38 | 7 |
| 28 |  | ko01250 | Biosynthesis of nucleotide sugars | 2.38% | 0.004 | 89 | 70 | 19 |
| 29 |  | ko00052 | Galactose metabolism | 1.98% | 0.004 | 74 | 48 | 26 |
| 30 |  | ko01240 | Biosynthesis of cofactors | 4.97% | 0.007 | 186 | 171 | 15 |
| 31 |  | ko00510 | N-Glycan biosynthesis | 1.1% | 0.007 | 41 | 40 | 1 |
| 32 |  | ko02010 | ABC transporters | 1.76% | 0.010 | 66 | 63 | 3 |
| 33 |  | ko00513 | Various types of N-glycan biosynthesis | 0.8% | 0.011 | 30 | 29 | 1 |
| 34 |  | ko00901 | Indole alkaloid biosynthesis | 0.21% | 0.013 | 8 | 7 | 1 |
| 35 |  | ko00941 | Flavonoid biosynthesis | 0.69% | 0.018 | 26 | 24 | 2 |
| 36 |  | ko00996 | Biosynthesis of various alkaloids | 0.11% | 0.019 | 4 | 1 | 3 |
| 37 |  | ko00360 | Phenylalanine metabolism | 0.83% | 0.020 | 31 | 23 | 8 |
| 38 |  | ko00310 | Lysine degradation | 1.15% | 0.020 | 43 | 39 | 4 |
| 39 |  | ko00965 | Betalain biosynthesis | 0.13% | 0.040 | 5 | 4 | 1 |
| 40 |  | ko00196 | Photosynthesis - antenna proteins | 0.51% | 0.043 | 19 | 18 | 1 |
| 1 | **4FF/8FF** | ko04141 | Protein processing in endoplasmic reticulum | 7.84% | 0.000 | 204 | 116 | 88 |
| 2 |  | ko01100 | Metabolic pathways | 47.89% | 0.000 | 1246 | 656 | 590 |
| 3 |  | ko00190 | Oxidative phosphorylation | 4.15% | 0.000 | 108 | 53 | 55 |
| 4 |  | ko01200 | Carbon metabolism | 7.61% | 0.000 | 198 | 105 | 93 |
| 5 |  | ko00020 | Citrate cycle (TCA cycle) | 2.11% | 0.000 | 55 | 29 | 26 |
| 6 |  | ko03010 | Ribosome | 6.96% | 0.000 | 181 | 86 | 95 |
| 7 |  | ko03050 | Proteasome | 2.46% | 0.000 | 64 | 31 | 33 |
| 8 |  | ko01040 | Biosynthesis of unsaturated fatty acids | 1.38% | 0.000 | 36 | 26 | 10 |
| 9 |  | ko01212 | Fatty acid metabolism | 3.07% | 0.000 | 80 | 47 | 33 |
| 10 |  | ko00250 | Alanine, aspartate and glutamate metabolism | 1.61% | 0.000 | 42 | 14 | 28 |
| 11 |  | ko00650 | Butanoate metabolism | 0.85% | 0.000 | 22 | 11 | 11 |
| 12 |  | ko00950 | Isoquinoline alkaloid biosynthesis | 1.19% | 0.000 | 31 | 16 | 15 |
| 13 |  | ko00052 | Galactose metabolism | 2.46% | 0.000 | 64 | 24 | 40 |
| 14 |  | ko04145 | Phagosome | 2.42% | 0.000 | 63 | 33 | 30 |
| 15 |  | ko00460 | Cyanoamino acid metabolism | 1.73% | 0.000 | 45 | 33 | 12 |
| 16 |  | ko00280 | Valine, leucine and isoleucine degradation | 2.04% | 0.000 | 53 | 24 | 29 |
| 17 |  | ko01110 | Biosynthesis of secondary metabolites | 26.71% | 0.000 | 695 | 388 | 307 |
| 18 |  | ko00360 | Phenylalanine metabolism | 1.11% | 0.000 | 29 | 8 | 21 |
| 19 |  | ko00073 | Cutin, suberine and wax biosynthesis | 0.96% | 0.000 | 25 | 15 | 10 |
| 20 |  | ko00440 | Phosphonate and phosphinate metabolism | 0.31% | 0.001 | 8 | 1 | 7 |
| 21 |  | ko00270 | Cysteine and methionine metabolism | 2.34% | 0.002 | 61 | 34 | 27 |
| 22 |  | ko00040 | Pentose and glucuronate interconversions | 2.27% | 0.003 | 59 | 33 | 26 |
| 23 |  | ko01230 | Biosynthesis of amino acids | 5.27% | 0.003 | 137 | 66 | 71 |
| 24 |  | ko00350 | Tyrosine metabolism | 1.23% | 0.003 | 32 | 13 | 19 |
| 25 |  | ko01210 | 2-Oxocarboxylic acid metabolism | 1.31% | 0.004 | 34 | 18 | 16 |
| 26 |  | ko00999 | Biosynthesis of various plant secondary metabolites | 1.69% | 0.005 | 44 | 35 | 9 |
| 27 |  | ko00600 | Sphingolipid metabolism | 1.27% | 0.005 | 33 | 11 | 22 |
| 28 |  | ko00901 | Indole alkaloid biosynthesis | 0.27% | 0.006 | 7 | 4 | 3 |
| 29 |  | ko00380 | Tryptophan metabolism | 1.42% | 0.008 | 37 | 19 | 18 |
| 30 |  | ko00053 | Ascorbate and aldarate metabolism | 1.84% | 0.009 | 48 | 25 | 23 |
| 31 |  | ko00513 | Various types of N-glycan biosynthesis | 0.88% | 0.009 | 23 | 14 | 9 |
| 32 |  | ko00450 | Selenocompound metabolism | 0.58% | 0.011 | 15 | 7 | 8 |
| 33 |  | ko00630 | Glyoxylate and dicarboxylate metabolism | 1.73% | 0.015 | 45 | 21 | 24 |
| 34 |  | ko00620 | Pyruvate metabolism | 2.61% | 0.017 | 68 | 37 | 31 |
| 35 |  | ko00062 | Fatty acid elongation | 0.73% | 0.018 | 19 | 10 | 9 |
| 36 |  | ko00604 | Glycosphingolipid biosynthesis - ganglio series | 0.58% | 0.019 | 15 | 3 | 12 |
| 37 |  | ko00640 | Propanoate metabolism | 1.31% | 0.020 | 34 | 18 | 16 |
| 38 |  | ko00410 | beta-Alanine metabolism | 1.31% | 0.025 | 34 | 19 | 15 |
| 39 |  | ko00944 | Flavone and flavonol biosynthesis | 0.15% | 0.026 | 4 | 3 | 1 |
| 40 |  | ko00511 | Other glycan degradation | 1.04% | 0.026 | 27 | 14 | 13 |
| 41 |  | ko00010 | Glycolysis / Gluconeogenesis | 3.31% | 0.032 | 86 | 45 | 41 |
| 42 |  | ko00943 | Isoflavonoid biosynthesis | 0.31% | 0.036 | 8 | 8 | 0 |
| 43 |  | Ko00510 | N-Glycan biosynthesis | 1.04% | 0.046 | 27 | 14 | 13 |
