# Supplementary material for: Transcriptome and metabolome profiling unveil the accumulation of chlorogenic acid in autooctoploid Gongju
Source: Front Plant Sci. 2024 Nov 1;15:1461357. doi: 10.3389/fpls.2024.1461357 (PMC11563975; doi:10.3389/fpls.2024.1461357)
Supplement: Supplementary file 15 [file Table7.docx]

**Table s7 Number of metabolites and differentially expressed genes during same flowering stages in tetraploid and octoploid gongju**

| **Grop** | **Number of metabolites** | **Number of genes** |
| --- | --- | --- |
| 4BS VS 8BS | 117 | 6837 |
| 4EF VS 8EF | 161 | 11211 |
| 4FF VS 8FF | 201 | 3859 |

Budding stage (BS), early flowering stage (EF) and full flowering stage (FF). 4 and 8: tetraploid and octoploid.
