# Supplementary material for: Transcriptome and metabolome profiling unveil the accumulation of chlorogenic acid in autooctoploid Gongju
Source: Front Plant Sci. 2024 Nov 1;15:1461357. doi: 10.3389/fpls.2024.1461357 (PMC11563975; doi:10.3389/fpls.2024.1461357)
Supplement: Supplementary file 16 [file Table8.docx]

**Table S8 Integration of metabolomics and transcriptome analysis for KEGG enrichmen**

| **4BS vs 8BS** | | |
| --- | --- | --- |
| Number | KEGG_map | Description |
| 1 | ko01100 | Metabolic pathways |
| 2 | ko01110 | Biosynthesis of secondary metabolites |
| 3 | ko00360 | Phenylalanine metabolism |
| 4 | ko00999 | Biosynthesis of various plant secondary metabolites |
| 5 | ko00380 | Tryptophan metabolism |
| 6 | ko00944 | Flavone and flavonol biosynthesis |
| 7 | ko00943 | Isoflavonoid biosynthesis |
| 8 | ko00960 | Tropane, piperidine and pyridine alkaloid biosynthesis |
| 9 | ko00940 | Phenylpropanoid biosynthesis |
| 10 | ko00330 | Arginine and proline metabolism |
| 11 | ko00902 | Monoterpenoid biosynthesis |
| 12 | ko00909 | Sesquiterpenoid and triterpenoid biosynthesis |
| 13 | ko00400 | Phenylalanine, tyrosine and tryptophan biosynthesis |
| 14 | ko00402 | Benzoxazinoid biosynthesis |
| **4EF vs 8EF** | | |
| 1 | ko01100 | Metabolic pathways |
| 2 | ko00943 | Isoflavonoid biosynthesis |
| 3 | ko00330 | Arginine and proline metabolism |
| 4 | ko00901 | Indole alkaloid biosynthesis |
| 5 | ko00941 | Flavonoid biosynthesis |
| 6 | ko00360 | Phenylalanine metabolism |
| 7 | ko00310 | Lysine degradation |
| 8 | ko00380 | Tryptophan metabolism |
| 9 | ko00940 | Phenylpropanoid biosynthesis |
| 10 | ko00480 | Glutathione metabolism |
| 11 | ko00350 | Tyrosine metabolism |
| 12 | ko00945 | Stilbenoid, diarylheptanoid and gingerol biosynthesis |
| 13 | ko00960 | Tropane, piperidine and pyridine alkaloid biosynthesis |
| 14 | ko00760 | Nicotinate and nicotinamide metabolism |
| 15 | ko00944 | Flavone and flavonol biosynthesis |
| 16 | ko00999 | Biosynthesis of various plant secondary metabolites |
| 17 | ko00902 | Monoterpenoid biosynthesis |
| 18 | ko00470 | D-Amino acid metabolism |
| 19 | ko00909 | Sesquiterpenoid and triterpenoid biosynthesis |
| 20 | ko01110 | Biosynthesis of secondary metabolites |
| **4FF vs 8FF** | | |
| 1 | ko00940 | Phenylpropanoid biosynthesis |
| 2 | ko00941 | Flavonoid biosynthesis |
| 3 | ko00945 | Stilbenoid, diarylheptanoid and gingerol biosynthesis |
| 4 | ko00943 | Isoflavonoid biosynthesis |
| 5 | ko00380 | Tryptophan metabolism |
| 6 | ko00902 | Monoterpenoid biosynthesis |
| 7 | ko01110 | Biosynthesis of secondary metabolites |
| 8 | ko00950 | Isoquinoline alkaloid biosynthesis |
| 9 | ko01100 | Metabolic pathways |
| 10 | ko00909 | Sesquiterpenoid and triterpenoid biosynthesis |
| 11 | ko00999 | Biosynthesis of various plant secondary metabolites |
| 12 | ko00360 | Phenylalanine metabolism |
| 13 | ko00350 | Tyrosine metabolism |
| 14 | ko00460 | Cyanoamino acid metabolism |
| 15 | ko00270 | Cysteine and methionine metabolism |
| 16 | ko00760 | Nicotinate and nicotinamide metabolism |
| 17 | ko00330 | Arginine and proline metabolism |
| 18 | ko01240 | Biosynthesis of cofactors |
